# Responses to Nei-Xiao-Luo-Li-San in Toledo Diffuse Large B-Cell Lymphoma Cells: Potential Involvement of ATF5- and HDAC-Associated Epigenetic Changes

**DOI:** 10.3390/biomedicines14092052

**Published:** 2026-09-12

**Authors:** Kaixuan Zou, Jong Hyuk Kim, Sahana Arunasalam, Betty Yangari, Deng-Shan Shiau

**Affiliations:** 1 Bioscience Research Center, Chi University, Reddick, FL 32686, USA; 2 Department of Small Animal Clinical Sciences, College of Veterinary Medicine, University of Florida, Gainesville, FL 32610, USA; 3 UF Health Cancer Institute, University of Florida, Gainesville, FL 32610, USA; 4 Artificial Intelligence Academic Initiative (AI2) Center, University of Florida, Gainesville, FL 32611, USA

**Keywords:** Diffuse large B-cell lymphoma, herbal formulation, histone deacetylase, ATF5, bioinformatics

## Abstract

**Background**: Diffuse large B-cell lymphoma (DLBCL) is the most common subtype of non-Hodgkin lymphoma in adults and is characterized by aggressive progression and substantial molecular and clinical heterogeneity. Despite advances in immunochemotherapy, a considerable proportion of patients develop relapsed or refractory disease, highlighting the need to better understand the molecular mechanisms underlying DLBCL. Nei-Xiao-Luo-Li-San (NXLLS), a traditional herbal formulation historically used for nodules and tumor-like masses, provides a pharmacological model for investigating molecular responses and candidate regulatory mechanisms in DLBCL. **Methods:** NXLLS was chemically characterized to assess its compositional profile and compositional similarity among batches. Its biological effects were evaluated by assessing cell viability, migration, invasion, and cell-cycle progression in Toledo DLBCL cells. Molecular responses to NXLLS were investigated through an integrated approach combining transcriptomic analysis, network pharmacology, and molecular assays. The functional role of ATF5 was further evaluated using lentiviral overexpression and knockdown. **Results:** Chromatographic profiling provided an exploratory assessment of compositional similarity among NXLLS samples and supported the selection of eight representative compounds using liquid chromatography–mass spectrometry (LC–MS) annotation and high-performance liquid chromatography (HPLC) authentic-standard confirmation. NXLLS reduced cell viability, migration, and invasion in Toledo DLBCL cells within the tested concentration range. At the molecular level, NXLLS was associated with altered cell-cycle distribution and potential G1-phase accumulation at later time points, reduced vascular endothelial growth factor (VEGF)-associated enzyme-linked immunosorbent assay (ELISA) signal, and modulated stress-response genes. RNA sequencing (RNA-seq) revealed transcriptional changes involving stress-response and histone deacetylase (HDAC)-associated epigenetic pathways. Correspondingly, HDAC expression was reduced, whereas acetylated histone H3 lysine 9 (Ac-H3K9) and acetylated histone H3 lysine 27 (Ac-H3K27) levels were increased. *ATF5* knockdown recapitulated several cellular and epigenetic changes observed following NXLLS treatment, supporting ATF5 as a potential downstream mediator of the NXLLS response. **Conclusions:** This study identified potential molecular targets associated with the response to NXLLS in Toledo DLBCL cells, with ATF5 further investigated as a potential downstream mediator. The findings also suggest an association between the NXLLS response and HDAC-related epigenetic changes, providing a basis for further investigation of their mechanistic relationships.

## 1. Introduction

Diffuse large B-cell lymphoma (DLBCL) is the most common subtype of non-Hodgkin lymphoma in adults, accounting for approximately 25 to 40% of all cases [1,2]. Its major clinical threat lies in its highly aggressive nature and rapid progression of the disease. Without treatment, the median survival time for patients with DLBCL has been reported to be less than a year [3]. Despite the curative potential of standard first-line immunochemotherapy regimens such as R-CHOP (Cyclophosphamide, doxorubicin, vincristine, and prednisone), approximately 30 to 50% of patients with DLBCL experience relapse or do not respond to treatment, resulting in refractory disease [4,5]. The prognosis for patients with relapsed or refractory DLBCL is generally poor, with a median overall survival of only 6.3 months and a 2-year survival rate of approximately 20% [5]. In particular, patients who relapse within one year of initial treatment face an even more challenging prognosis [4].

DLBCL is characterized by a high degree of clinical, morphological, and genetic heterogeneity [6], which underlies the wide variation in survival outcomes observed among patients [7]. For instance, gene expression profiling has enabled the classification of DLBCL into two principal molecular subtypes: germinal center B-cell-like (GCB) and activated B-cell-like (ABC). These subtypes differ substantially in their pathogenesis and response to standard chemotherapy regimens, with the ABC subtype generally associated with inferior clinical outcomes [8,9]. In addition, conventional therapeutic modalities such as chemotherapy, despite their efficacy in eliminating malignant cells, often cause collateral damage to normal tissues and organs [10]. Common adverse effects include neutropenia, nausea, and diarrhea, while more serious complications, including cardiotoxicity and increased susceptibility to infections can also arise [11]. These limitations underscore the urgent need to deepen our mechanistic understanding of DLBCL, which may inform the development of more targeted and precise therapeutic strategies capable of improving patient outcomes while minimizing treatment-related complications [12].

Traditional Chinese medicine (TCM) has demonstrated preventive and therapeutic effects against DLBCL, with a favorable side-effect profile that represents a notable clinical advantage [13,14]. Nei-Xiao-Luo-Li-San (NXLLS) is a cancer-targeting herbal formulation [15], derived from the classical Qing dynasty TCM formulation Nei-Xiao-Luo-Li-Wan, originally recorded in Gu Shicheng’s *Complete Compendium of Ulcer Medicine* (*Yang Yi Da Quan*). Its name reflects its traditional therapeutic concept, in which nei-xiao denotes the internal resolution or dissipation of pathological nodular masses. In TCM, Luo-Li refers to small, palpable nodular masses, most commonly occurring in the cervical region and associated with chronic lymphatic involvement [16]. Clinically, these manifestations correspond to enlarged lymph nodes and mass-forming lesions, which share notable symptomatic similarities with lymphoid malignancies such as lymphoma. The traditional indications of Nei-Xiao-Luo-Li-Wan for conditions categorized as Luo-Li therefore provide an ethnopharmacological basis to explore its potential relevance in modern lymphatic neoplastic disorders. However, Nei-Xiao-Luo-Li-Wan showed limited efficacy when used as a comparator treatment in a clinical study of benign uterine leiomyomata (rather than malignant disease) [17], and its traditional formulation presents challenges related to standardization and quality control [18]. Accordingly, modernization and reformulation are considered essential steps toward optimizing its clinical utility.

Nei-Xiao-Luo-Li-San (NXLLS) is the powdered form of the original pill formulation and has been used in clinical practice for over two decades. Compared to the original formulation, the mineral components, including sodium nitrate and processed salt, as well as the excipients associated with the pill dosage form, were excluded, while the remaining constituents were preserved [15]: *Prunella vulgaris*, *Magallana gigas*, *Fritillaria thunbergii*, *Trichosanthes kirilowii*, *Scrophularia ningpoensis*, *Platycodon grandiflorus*, *Angelica dahurica*, and *Rheum palmatum*. Although these constituents have not been specifically studied in the context of DLBCL, many have demonstrated anti-cancer activity across a range of malignancies, suggesting their potential therapeutic relevance and warranting further investigation. For example, *Prunella vulgaris* showed antiproliferative effects in breast cancer cells [19], while *Fritillaria thunbergii* and *Trichosanthes kirilowii* alkaloids induced apoptosis in lung and cervical cancer models, respectively [20,21]. *Scrophularia ningpoensis* and *Rheum palmatum* have been reported to inhibit the growth of colon and liver cancer cells through apoptosis-related pathways [22,23]. Moreover, compounds from *Angelica dahurica* and *Platycodon grandiflorus* have shown modest antioxidant or anticancer effects in colon cancer cells and lung cells [24,25,26].

In this study, we comprehensively characterized the chemical profile of this multi-herb formulation NXLLS and investigated its biological effects in Toledo DLBCL cells to elucidate its molecular mechanisms and facilitate the identification of novel therapeutic targets for this malignancy.

## 2. Materials and Methods

### 2.1. Preparation of NXLLS

The NXLLS powder used in this study was manufactured by Jiangyin Tianjiang Pharmaceutical Co., Ltd., Jiangyin, China from eight medicinal materials (Table 1), manufacturing followed standardized procedures in accordance with the *Pharmacopoeia of the People’s Republic of China*. These procedures included raw material inspection, aqueous extraction at 100 °C, low-temperature concentration, spray drying, and granulation. Independent STR profiling and mycoplasma testing were not performed for the cell lines used in this study.

Following receipt, the standardized NXLLS powder was kept in a sealed container at 4 °C until use, and laboratory extracts were freshly prepared for the experiments. Production batch no. 907272000027 was used for the majority of experiments throughout the study. Batch-to-batch consistency was evaluated by HPLC fingerprint similarity analysis using two additional production batches (batch nos. 904212200012 and 901072000018). For extract preparation, 2 g of granulated NXLLS powder was added to 10 mL of each of the following four vehicles: 60% ethanol (EtOH), 100% dimethyl sulfoxide (DMSO), 0.1 N hydrochloric acid (HCl), and phosphate-buffered saline (PBS). The powder was dissolved in separate 50 mL centrifuge tubes, thoroughly vortexed, and placed in a shaking incubator at 24 °C, 60 rpm to facilitate dissolution. Extracts prepared in 60% EtOH and 100% DMSO were incubated for 3 days, while those prepared in 0.1 N HCl and PBS were incubated for 1 day.

After incubation, the samples were centrifuged at 1500 × g for 10 min to separate the supernatant from the insoluble material. The supernatant was collected and filtered through a 0.22 µm sterile pore-size filter unit, transferred to microcentrifuge tubes and aliquoted into 100 µL portions to minimize repeated freeze–thaw cycles. The aliquots were stored at −80 °C until use. Extraction yield was not determined; therefore, the reported NXLLS concentrations refer to the mass of starting granulated powder per unit volume rather than the measured concentration of dissolved solids.

### 2.2. Thin-Layer Chromatography Analysis

Prior to NXLLS preparation, all crude medicinal materials listed in Table 1 were authenticated by thin-layer chromatography (TLC) through comparison with the corresponding reference standards. Samples and reference standards were applied to silica gel TLC plates and developed using optimized mobile phase systems selected according to the chemical characteristics of each material. After development, the plates were air-dried and visualized under ultraviolet light at 254 and/or 365 nm.

### 2.3. High-Performance Liquid Chromatography (HPLC) Fingerprint Analysis

HPLC analysis was performed on a DW-LC1620A liquid chromatography system (Chongqing Drawell Instrument Co., Ltd., Chongqing, China) using a SymmetryShield RP8 column (4.6 × 250 mm, 5 µm; Waters Corporation, Milford, MA, USA). The mobile phase was a mixture of solvent A (water containing 0.1% formic acid) and solvent B (acetonitrile), delivered using the following gradient elution program: 0–12 min, 97:3 (A:B); 12–28 min, linear gradient from 97:3 to 90:10; 28–45 min, linear gradient from 90:10 to 70:30; 45–60 min, linear gradient from 70:30 to 40:60; 60–70 min, linear gradient from 40:60 to 10:90; 70–75 min, held at 10:90; 75–77 min, returned to 97:3; and 77–90 min, held at 97:3 for column re-equilibration. The flow rate was set at 1.0 mL/min, the column temperature was maintained at 30 °C, and the injection volume was 5 µL. Detection was carried out using a UV detector at 280 nm, with additional monitoring at 254 nm.

For chromatographic peak identification, three representative compounds from the LC–MS profiling results were selected for confirmation using authentic reference standards. Individual standards were first analyzed to determine the retention time (RT) of each compound, followed by analysis of mixed standards to confirm the absence of peak overlap. NXLLS samples and NXLLS samples spiked with mixed standards were subsequently analyzed to verify the presence and enhancement of the corresponding peaks. Detection was carried out using a UV detector at 360 nm, with additional monitoring at 280 nm, and the injection volume was 5 µL. Authentic standards of quercetin (Cat. No. HY-18085), kaempferol (Cat. No. HY-14590) and rutin (Cat. No. HY-N0148) were purchased from MedChemExpress LLC, Monmouth Junction, NJ, USA. Stock solutions (10 mM) were prepared in DMSO and diluted 200-fold in 50% methanol prior to HPLC analysis. Chromatographic data were processed using the Similarity Evaluation System for the TCM chromatographic fingerprint (Version 2012.130723; Chinese Pharmacopoeia Commission, Beijing, China). The similarity between different samples was calculated on the basis of the correlation coefficient method after alignment and normalization of the chromatogram. Chromatographic figures were generated using Origin (Version 10.2.5.212; OriginLab Corporation, Northampton, MA, USA).

### 2.4. Liquid Chromatography–Mass Spectrometry (LC–MS) Analysis

The chemical composition of NXLLS was characterized by LC–MS at the Mass Spectrometry Research and Education Center, University of Florida. The ethanolic extract was diluted 1:1 (*v*/*v*) with methanol before analysis. Chromatographic separation was performed on a ProntoSIL C18AQ column (300 µm × 15 cm, 3 µm) using an UltiMate 3000 LC system (Thermo Fisher Scientific Inc., Waltham, MA, USA) with a 60 min gradient of water containing 10 mM ammonium formate and 0.1% formic acid and acetonitrile containing 0.1% formic acid.

Mass spectrometric analysis was conducted on a Bruker timsTOF Pro II (Bruker Daltonics GmbH & Co. KG, Bremen, Germany) instrument equipped with a heated electrospray ionization source and operated in both positive and negative ion modes. Data were processed using Bruker DataAnalysis (Version 6.0; Bruker Daltonics GmbH & Co. KG, Bremen, Germany). and MetaboScape (Version 2024b; Bruker Daltonics GmbH & Co. KG, Bremen, Germany). Features were putatively annotated through accurate-mass, isotope-pattern, and MS/MS spectral-library matching using mass tolerances of up to 5 ppm. Features without spectral matches were assigned candidate molecular formulas using SmartFormula. Compound annotations were considered putative unless independently confirmed using authentic reference standards.

### 2.5. Cell Culture and NXLLS Treatment

The human DLBCL cell line Toledo (ATCC, CRL-2631^TM^), the T-cell lymphoblastic lymphoma (T-LBL) cell line SUP-T1 (ATCC, CRL-1942^TM^) and the mixed primary human renal epithelial cell line HREC (ATCC, PCS-400-012^TM^) were purchased from ATCC. The human esophageal squamous cell carcinoma line KYSE-520 (Research Resource Identifier [RRID]: CVCL 1355) was obtained from the laboratory of Dr. Takayoshi Tobe (Kyoto University, Kyoto, Japan). All cell lines were cultured according to the suppliers’ recommended protocols. Specifically, Toledo, SUP-T1 and KYSE-520 cells were maintained in RPMI-1640 medium (ATCC, 30-2001^TM^). HREC cells were cultured in basal medium for renal epithelial cells (ATCC, PCS-400-030^TM^) supplemented with the recommended growth kit (ATCC, PCS-400-040^TM^). All media were supplemented with 10% fetal bovine serum (FBS), 100 units/mL penicillin G, 100 µg/mL streptomycin sulfate and 0.25 µg/mL amphotericin B. The cells were incubated at 37 °C in a humidified incubator with 5% CO_2_. Toledo cells were maintained at a density between 3 × 10^5^ and 3 × 10^6^ viable cells/mL, and SUP-T1 cells between 2 × 10^5^ and 2 × 10^6^ viable cells/mL. HREC and KYSE-520 cells were passaged when they reached approximately 70% confluency.

The effects of the NXLLS extracts and chemotherapy agents on cell viability were evaluated and compared across different cell lines. Toledo and SUP-T1 cells were seeded in 96-well plates at a density of 1 × 10^4^/well with 100 µL corresponding culture medium, while KYSE-520 and HREC cells were seeded at a density of 4 × 10^3^/well. Countess^TM^ 3 FL Automated Cell Counter (Thermo Fisher Scientific Inc., Waltham, MA, USA) was used for automatic cell counting. Cyclophosphamide (Cat. No. HY-17420), doxorubicin hydrochloride (Standard; Cat. No. HY-15142R), prednisone (Cat. No. HY-B0214), and vincristine sulfate (Cat. No. HY-N0488) were purchased from MedChemExpress (Monmouth Junction, South Brunswick, NJ, USA). An 8-point 2-fold serial dilution of NXLLS extracts or chemotherapy medicines was performed horizontally across eight columns of a 96-well plate. Each concentration was applied in triplicate in the vertical direction in three rows. Three additional rows were treated with the corresponding vehicle control in the same volume as the medicine-treated wells. The final column of the plate was left untreated as an untreated control without any treatment. Cells were incubated with the indicated treatments for 72 h before cell viability was assessed. The maximum final treatment concentrations were 25 mg/mL for NXLLS, 100 µM for cyclophosphamide, 10 µM for doxorubicin, 30 µM for vincristine, 300 µM for prednisone, 25 µM for cediranib, and 25 µg/mL for puromycin. Serial dilutions were prepared from these concentrations, with additional lower concentrations tested where necessary to adequately cover the dose–response range. Because NXLLS is a multi-component herbal formulation and its molecular weight cannot be accurately determined, both NXLLS and the chemotherapy agents were converted to mass-based units for comparison.

### 2.6. Cell Viability Assay

The cell viability assay was performed using WST-1 assay (2-(4-iodophenyl)-3-(4-nitrophenyl)-5-(2,4-disulfophenyl)-2H-tetrazolium monosodium salt), a colorimetric assay that measures the metabolic activity of viable cells. After treatment, 10 µL of WST-1 reagent (Dojindo Laboratories Co., Ltd., Kumamoto, Japan) was added to each well of a 96-well plate, followed by incubation for 30 min. Optical density (OD) values at 450 nm and 610 nm were measured at 30 min intervals using a microplate reader, and the measurements continued until stable absorbance values were obtained. The OD value at 610 nm was used as a background reference. Cell viability was calculated by subtracting the OD value at 610 nm from that at 450 nm. Relative cell viability rate was calculated as a percentage of the vehicle-treated control group. The inhibition rate was calculated by subtracting the viability rate from 100%, with higher inhibition rates indicating greater reductions in cell viability and potential cytotoxic effects. Dose–response curves were generated, and the half-maximum inhibitory concentration (IC_50_) was determined using GraphPad Prism (version 10.1.2; GraphPad Software, Boston, MA, USA). IC_50_ values were used as a quantitative measure of treatment potency, enabling comparisons of the relative inhibitory effects of different compounds on cancer cell viability, with lower IC_50_ values indicating greater potency.

### 2.7. RNA Sequencing (RNA-seq) and Preprocessing

Total RNA was extracted from NXLLS-treated and vehicle-treated Toledo cells using AurumTM Total RNA Mini Kit (Bio-Rad Laboratories, Inc., Hercules, CA, USA) according to the manufacturer’s instructions. Toledo cells were treated with EtOH-extracted NXLLS at its IC_50_ concentration or the corresponding vehicle control for 72 h. The IC_50_ concentration was selected based on the cell viability assay described above. A total of six RNA-seq samples were generated, including three biological replicates per experimental condition, with 1 × 10^6^ cells used for each sample. 2 µg of RNA was submitted to the Genomics Shared Resources at Roswell Park Comprehensive Cancer Center (Buffalo, NY, USA) for library preparation. Sequencing was performed as paired-end libraries on the Illumina platform (flow cell HHJMLDRX2), with a target sequencing depth of approximately 20–30 million paired-end reads per sample. The Genomics Shared Resource performed the initial bioinformatic processing. Raw sequencing reads were initially assessed for quality using FastQC. Splice-aware alignment to the human reference genome GRCh38.p13 was performed using STAR, with gene annotations from GENCODE release 38 (December 2020). Post-alignment quality control was conducted with RSeQC to assess read distribution and mapping quality. Gene-level read counts were generated using featureCounts from the Subread package (version 2.1.1), with fracOverlap = 1.

### 2.8. Bioinformatics Analysis

Differentially expressed genes (DEGs) between the NXLLS-treated and vehicle-treated groups was also performed by the Genomics Shared Resource using the DESeq2 package (version 1.36.0) under R 4.2/Bioconductor 3.15, using the Wald test implemented in DESeq2. The authors subsequently used the facility-generated differential expression results for downstream analysis in R (version 4.5.0). Differentially expressed genes were selected using an absolute log2 fold change ≥ 1 and a Benjamini–Hochberg-adjusted *p* value < 0.05. Pathway-enrichment analysis and data visualization, including the volcano and bubble plots, were performed by the authors.

Representative compounds putatively annotated from the LC–MS profile and assigned PubChem compound identifiers were submitted to BATMAN-TCM (Version 1.0) to retrieve known and predicted compound–target associations. The analysis was performed using the default parameters of the BATMAN-TCM platform. Compound–target associations returned under these default settings were retained for subsequent analyses. The retained targets were combined and deduplicated before exploratory GO, KEGG, OMIM, and TTD enrichment analyses. Enriched terms with Benjamini–Hochberg-adjusted *p* values < 0.05 were retained and visualized in R. These prediction-based analyses were used only for descriptive visualization and hypothesis generation and were not interpreted as evidence of pharmacological mechanism or causal relationships.

Gene Ontology (GO), Kyoto Encyclopedia of Genes and Genomes (KEGG), and Gene Set Enrichment Analysis (GSEA) were performed using the clusterProfiler package (version 4.16.0). GO and KEGG terms with adjusted *p* values < 0.05 were considered significantly enriched. For GSEA, pathways with BH-adjusted *p* values < 0.05 were considered significantly enriched. The Reactome database (accessed on 1 May 2025) was included because of its high-quality, manually curated pathway annotations, particularly for biological processes such as chromatin remodeling, DNA repair, and cell cycle regulation. Enrichment analyses based on online Mendelian inheritance in man (OMIM, https://www.omim.org/; accessed on 1 May 2025) and the Therapeutic Target Database (TTD, http://db.idrblab.net/ttd/; accessed on 1 May 2025) were also performed using curated gene sets downloaded from their respective official websites. Disease-associated terms from OMIM and TTD with adjusted *p* values < 0.05 were also summarized for descriptive reference.

Various R packages were used for data visualization and performing relevant analyzes. Specifically, the ggplot2 package (version 3.5.1), scales package (version 1.3.0), showtext package (version 0.9-6), latex2exp package (version 0.9.6), and ggrepel package (version 0.9.5) were used for general plotting and graphical enhancements, including bubble plots, volcano plot.

Kaplan–Meier survival analysis was performed using RNA-seq gene expression and clinical data from the TCGA-DLBC cohort. RNA-seq data processed using the STAR workflow were obtained from The Cancer Genome Atlas (TCGA) database [27] through the Genomic Data Commons (GDC). TPM-formatted expression data were extracted and log2(value + 1)-transformed for analysis. After excluding normal samples and samples without available clinical information, a total of 48 DLBCL samples were included. Patients were stratified into high- and low-expression groups for each gene based on the median expression level of the corresponding gene. Survival analysis was performed using the survminer package (version 0.4.9) in conjunction with the survival package (version 3.3.1), and Kaplan–Meier survival curves were compared using the log-rank (Mantel–Cox) test.

For ROC (Receiver operating characteristic) analysis, uniformly processed RNA-seq expression data were obtained through the UCSC Xena Toil (accessed on 1 May 2025) RNA-seq Recompute Compendium [28]. The dataset contained 491 samples, comprising 47 TCGA-DLBC primary tumor samples, 337 GTEx whole-blood samples, and 107 GTEx Epstein–Barr virus (EBV)-transformed lymphocyte samples. No TCGA adjacent normal samples were available. All TCGA and GTEx RNA-seq data had been processed using the same Toil workflow and expressed as transcripts per million (TPM), followed by log2(value + 1) transformation. ROC curves and area under the curve (AUC) values were calculated using the pROC package (version 1.18.0). The 95% confidence intervals for the AUC values were estimated using DeLong’s method. Because the GTEx reference group consisted of unmatched whole-blood samples and EBV-transformed lymphocyte cell lines, this analysis was treated as an exploratory, descriptive comparison of *ATF5* expression between TCGA-DLBC tumors and GTEx reference samples and was not interpreted as evidence of clinical diagnostic performance.

Additional data manipulation and input handling were performed using the readxl package (version 1.4.3), dplyr package (version 1.1.4), and stringr package (version 1.5.1).

### 2.9. Quantitative Real-Time PCR (RT-qPCR)

To assess *NEAT1* transcript expression following NXLLS treatment, RT-qPCR was performed using an independent set of cells from those used for RNA-seq. Total RNA was extracted using the Aurum™ Total RNA Mini Kit (Bio-Rad). RNA concentration was measured using a NanoDrop™ spectrophotometer (Thermo Fisher Scientific Inc., Waltham, MA, USA), and purity was assessed using the A260/A280 ratio, with values between 1.8 and 2.0 considered acceptable. Total RNA was reverse-transcribed into cDNA using the iScript™ cDNA Synthesis Kit (Bio-Rad Laboratories, Inc., Hercules, CA, USA), and RT-qPCR was performed using SsoAdvanced™ Universal SYBR^®^ Green Supermix (Bio-Rad) on a CFX Opus 96 Real-Time PCR System (Bio-Rad). Commercially available pre-designed Bio-Rad PrimePCR assays were used for human *NEAT1* (Entrez Gene ID: 283131) and the reference gene TUBB6 (Entrez Gene ID: 203068). The proprietary primer sequences were not disclosed by the manufacturer. All procedures were performed according to the manufacturer’s instructions. Relative *NEAT1* expression was calculated using the 2^−ΔΔ*Cq*^ method, with TUBB6 as the reference gene and the vehicle-treated group as the calibrator. Data were analyzed using Bio-Rad CFX Maestro software and GraphPad Prism.

### 2.10. Western Blot

Western blot experiments were conducted using the Toledo cell line. For dose-dependent experiments, cells were treated with NXLLS at concentrations of 0.25 × IC_50_, 0.5 × IC_50_, 1 × IC_50_, 2 × IC_50_, and 4 × IC_50_ for 72 h. For time-dependent experiments, cells were treated with NXLLS at the IC_50_ concentration and harvested at various time points up to 72 h. Based on preliminary experiments, subsequent histone acetylation analyses were performed after 24 h of treatment.

The total protein was extracted using RIPA lysis buffer (Bio-Rad), while histones were extracted with the Histone Extraction Kit (ab113476; Abcam, Waltham, MA, USA). Protein concentrations were determined using the BCA assay. The composition of the hand-cast gels followed the Bio-Rad hand-casting polyacrylamide gel protocol (Bulletin 6201). In the section involving lentiviral infection, 4–20% Mini-PROTEAN^®^ TGX^TM^ Precast Protein Gels (Bio-Rad, 4561093) were used instead of hand-cast gels. Western blot imaging was performed using the Odyssey Fc Imager (LI-COR). Band intensities were quantified with ImageJ software (version 1.53k; National Institutes of Health, Bethesda, MD, USA) and statistical analysis was performed using GraphPad Prism. Protein expression levels were calculated as the ratio of the intensity of the target band to that of the internal control. Details of the antibodies used are provided in Table S1.

### 2.11. Indirect Enzyme-Linked Immunosorbent Assay (ELISA)

VEGF-associated signals in the culture supernatants were assessed using an indirect ELISA. Briefly, 100 µL of culture supernatant was added to each well of a 96-well microplate and incubated overnight at 4 °C to allow adsorption of soluble proteins. After washing and blocking, the wells were incubated with a mouse monoclonal anti-VEGF primary antibody (Cat. No. sc-7269; Santa Cruz Biotechnology, Inc., Dallas, TX, USA) at a 1:500 dilution. The plates were then washed and incubated with an HRP-conjugated anti-mouse secondary antibody (#31430; Thermo Fisher Scientific Inc., Waltham, MA, USA) at a 1:10,000 dilution. Color development was performed using Invitrogen™ ABTS substrate solution (Cat. No. 002024). Absorbance was measured at 410 nm using a microplate reader. Background correction was performed by subtracting the OD_410_ value of wells processed identically but without cell culture supernatant from the corresponding sample value. The resulting blank-corrected OD_410_ values were used for subsequent analysis.

### 2.12. Transwell Cell Migration and Invasion Assay

To evaluate cell migration and invasion, Transwell assays were performed using 6-well hanging cell culture inserts. For the migration assay, cells were first treated with NXLLS at its concentration of IC_50_ for 3 days, followed by incubation with 1 µg/mL mitomycin for 2 h to inhibit proliferation. Subsequently, 3 × 10^6^ viable cells were seeded in each 5.0 µm pore-size insert (9305012; cellQART, SABEU GmbH & Co. KG, Northeim, Germany) containing 3 mL of serum-free RPMI-1640 medium. The inserts were then placed in 6-well plates containing 3 mL of RPMI-1640 supplemented with 10% FBS as a chemoattractant. After 24 h of incubation, the inserts were removed and the cells that migrated/invaded to the lower chamber were quantified using a WST–1-based cell viability assay. For the invasion assay, 8.0 µm pore-size inserts (9308012; cellQART, SABEU GmbH & Co. KG, Northeim, Germany) were precoated with 2% Matrigel Matrix (Cat. No. 356234; Corning Incorporated, Corning, NY, USA) for 2 h at 37 °C before cell seeding. The rest of the procedure was identical to that of the migration assay.

To estimate the number of migrated or invaded cells, a standard curve (linear regression model) was established to estimate the number of Toledo cells (in 3 mL of medium per well) from the corresponding WST-1 OD values. The OD values, which were measured after 3 h of WST-1 incubation, from the experimental wells were then applied to this regression model to estimate the total number of migrated or invaded cells. Migration or invasion rate was expressed as the percentage of viable cells that traversed the membrane in relation to the total number of input cells.

### 2.13. Flow Cytometry

To evaluate the effect of NXLLS on cell cycle progression, Toledo cells were treated with NXLLS at the IC_50_ concentration for 12, 24, 36 and 48 h. Cells treated with an equal volume of EtOH vehicle for 48 h served as the vehicle control. After treatments, cells were harvested and washed once with PBS, then fixed dropwise with pre-chilled 70% EtOH (−20 °C) overnight. Following fixation, cells were washed twice with PBS to remove residual EtOH. Approximately 1 × 10^6^ cells were resuspended in 0.5 mL of FxCycle^TM^ Propidium Iodide/RNase Staining Solution (F10797; Thermo Fisher Scientific Inc., Waltham, MA, USA) and incubated at room temperature for 30 min in the dark. Samples were analyzed at the UF Interdisciplinary Center for Biotechnology Research using a BD LSR Fortessa flow cytometer (BD Biosciences, San Jose, CA, USA) equipped with a 488 nm excitation laser and a 585/42 nm bandpass emission filter. Cell cycle distribution (G_1_, S, G_2_ phases) was determined based on DNA content using FlowJo (version 10.8.1). Flow-cytometric analysis was performed as a single descriptive experiment without independent biological replication; therefore, no inferential statistical analysis was conducted.

The proliferation index (PI) was calculated as a quantitative indicator of cell-cycle progression and proliferative activity, reflecting the proportion of cells actively undergoing DNA synthesis and mitosis. PI was calculated using the following formula:

$$PI=\frac{S+G_{2}}{G_{1}+S+G_{2}}\times 100\%$$

### 2.14. Transduction

To manipulate *ATF5* expression, cells were transduced with lentiviral particles for *ATF5* overexpression or short hairpin RNA (shRNA) mediated knockdown, followed by puromycin selection. Lentiviral vectors targeting *ATF5* were obtained from VectorBuilder Inc., Chicago, IL, USA. For overexpression, cells were transduced with a lentiviral construct encoding full-length *ATF5* (Vector ID: VB900011-1060msp) and a corresponding empty vector was used as a control (Vector ID: VB010000-9389rbj). For knockdown, cells were transduced with a lentivirus expressing *ATF5*-targeted shRNA (Vector ID: VB900085-9017xas), with a scrambled shRNA vector serving as a control (Vector ID: VB010000-0009mxc). Before transduction, a puromycin killing curve was performed to determine the optimal selection concentration. In addition, a multiplicity of infection (MOI) optimization assay was performed to optimize transduction efficiency using a control empty vector. Cells were transduced at the determined MOI and selected with puromycin to establish stable cell lines. Detailed procedures for establishing stable lentivirally transduced cell lines are provided in Supplementary Document S1. Following lentiviral transduction, viable cells were isolated using Lympholyte-H Cell Separation Media (Cat. No. CL5020; CEDARLANE Corporation, Burlington, ON, Canada) prior to subsequent experiments.

### 2.15. Statistical Analysis

Statistical analyses were performed using GraphPad Prism and R (version 4.5.0). Experiments involving two independent variables, such as drug treatment studies evaluating the effects of concentration and treatment duration, were analyzed using two-way analysis of variance (ANOVA), followed by Šídák’s multiple comparisons test for prespecified comparisons. One-way ANOVA was used to assess differences among three or more groups, including qPCR, and Transwell migration and invasion assays, and Western blot analysis of the lentiviral *ATF5* overexpression and knockdown experiments. For qPCR analyses involving multiple treatment concentrations or time points, Dunnett’s multiple comparisons test was used to compare each treatment group with the corresponding control group. For the Transwell assays and Western blot analysis of the lentiviral transduction experiments, Šídák’s multiple comparisons test was used for prespecified group comparisons.

Unless otherwise specified, quantitative experiments were performed using three independent biological replicates.

## 3. Results

### 3.1. Chemical Profiling, Chromatographic Similarity, and Representative Compound Identification in NXLLS

We first characterized the chemical profile of NXLLS using complementary chromatographic approaches, including TLC, HPLC and LC–MS. The herbal preparation was subjected to quality-control assessment, including appearance evaluation, moisture content determination, solubility testing, microbial limit testing, and heavy metal analysis. All parameters met the standards stipulated by the Chinese Pharmacopeia and the manufacturer’s specifications (Supplementary Document S2), providing preliminary quality-control information for the formulation used in this study.

**Figure 1:**
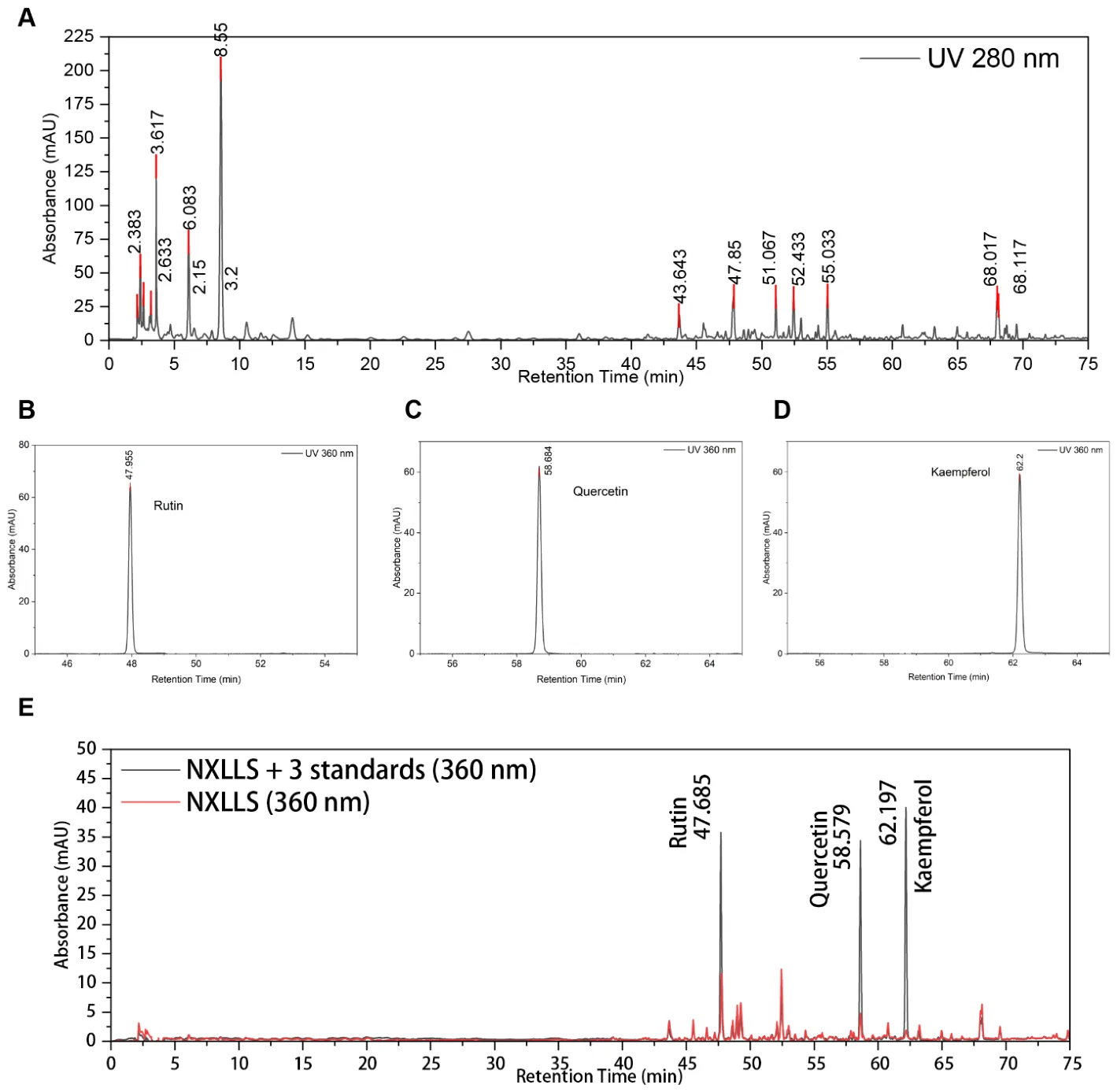
HPLC fingerprinting and targeted verification of representative compounds in NXLLS. (**A**) Representative HPLC chromatographic fingerprint of NXLLS recorded at 280 nm. Multiple characteristic peaks were observed across the chromatographic profile, with several prominent peaks detected in the early retention region (2–10 min). Additional characteristic peaks were also observed in the late retention region (43–68 min), illustrating the chemical complexity of the NXLLS extract. (**B**–**D**) HPLC chromatograms of individual reference standards recorded at 360 nm, showing the retention times of rutin (47 min), quercetin (58 min), and kaempferol (62 min), respectively. (**E**) Overlay chromatograms of NXLLS extract and NXLLS spiked with mixed standards at 360 nm. Corresponding peaks matching the retention times of rutin, quercetin, and kaempferol were observed in the NXLLS sample, while standard addition increased the intensities of the corresponding pre-existing peaks without producing additional peaks, further supporting the chromatographic identification of these compounds by authentic-standard comparison and HPLC co-injection.

TLC analysis demonstrated that the medicinal materials used for preparation of the NXLLS in this study were consistent with their corresponding reference standards (Supplementary Document S3), supporting the identity of the raw materials examined. While a primary NXLLS production batch was used for the majority of experiments in this study, 2 additional production batches were analyzed by HPLC fingerprint similarity analysis to assess chromatographic similarity among batches. The overall similarity of the characteristic peaks indicated broadly comparable chromatographic profiles across the three analyzed batches, although some inter-batch variation remained (Supplementary Document S4). A representative HPLC chromatographic fingerprint recorded at 280 nm is shown in Figure 1A.

Our data showed multiple well-resolved peaks predominantly within the retention window of 2–15 min, with a dominant peak observed at approximately 8.55 min. To further evaluate chromatographic reproducibility, additional NXLLS batches were independently analyzed by HPLC. The analysis showed a mean intra-batch similarity of 0.967 and a mean inter-batch similarity of 0.929, indicating high overall chromatographic similarity within batches while also revealing some inter-batch variation. LC–MS profiling yielded 142 putatively annotated features representing major phytochemical classes such as flavonoids, phenolic compounds, alkaloids, and fatty acids, providing a preliminary overview of the chemical composition of NXLLS. Based on feature traceability, signal quality, annotation evidence, and chemical representativeness, 8 representative compounds were retained for subsequent exploratory analyses (Table 2). 5 compounds were putatively annotated by LC–MS, whereas quercetin, kaempferol, and rutin were further evaluated using authentic HPLC standards and co-injection. Supplementary Document S5 contains the complete LC–MS feature export, including the bucket label, retention time, observed m/z, compound name, molecular formula, sample signal intensity, and blank-control signal intensity. The 8 features retained in Table 2 correspond to rows 3, 93, 130, 135, 137, 139, 1477, and 1478 of Supplementary Document S5.

Based on candidate LC–MS features with compatible m/z values and molecular formulas, quercetin, kaempferol, and rutin were further evaluated using authentic reference standards by HPLC. The standards were individually analyzed under the same chromatographic conditions used for the NXLLS sample, with rutin, quercetin, and kaempferol eluting at approximately 47, 58, and 62 min, respectively (Figure 1B–D). A mixture of the three standards was subsequently analyzed to verify chromatographic separation and the absence of peak overlap. The NXLLS sample exhibited peaks at the corresponding retention times. Moreover, spiking the NXLLS sample with the mixed standards increased the intensities of the corresponding pre-existing peaks without producing additional peaks (Figure 1E), supporting the presence and chromatographic identification of rutin, quercetin, and kaempferol in NXLLS by authentic-standard comparison and HPLC co-injection.

### 3.2. Exploratory Functional Enrichment Analyses of Representative NXLLS Compounds

**Figure 2:**
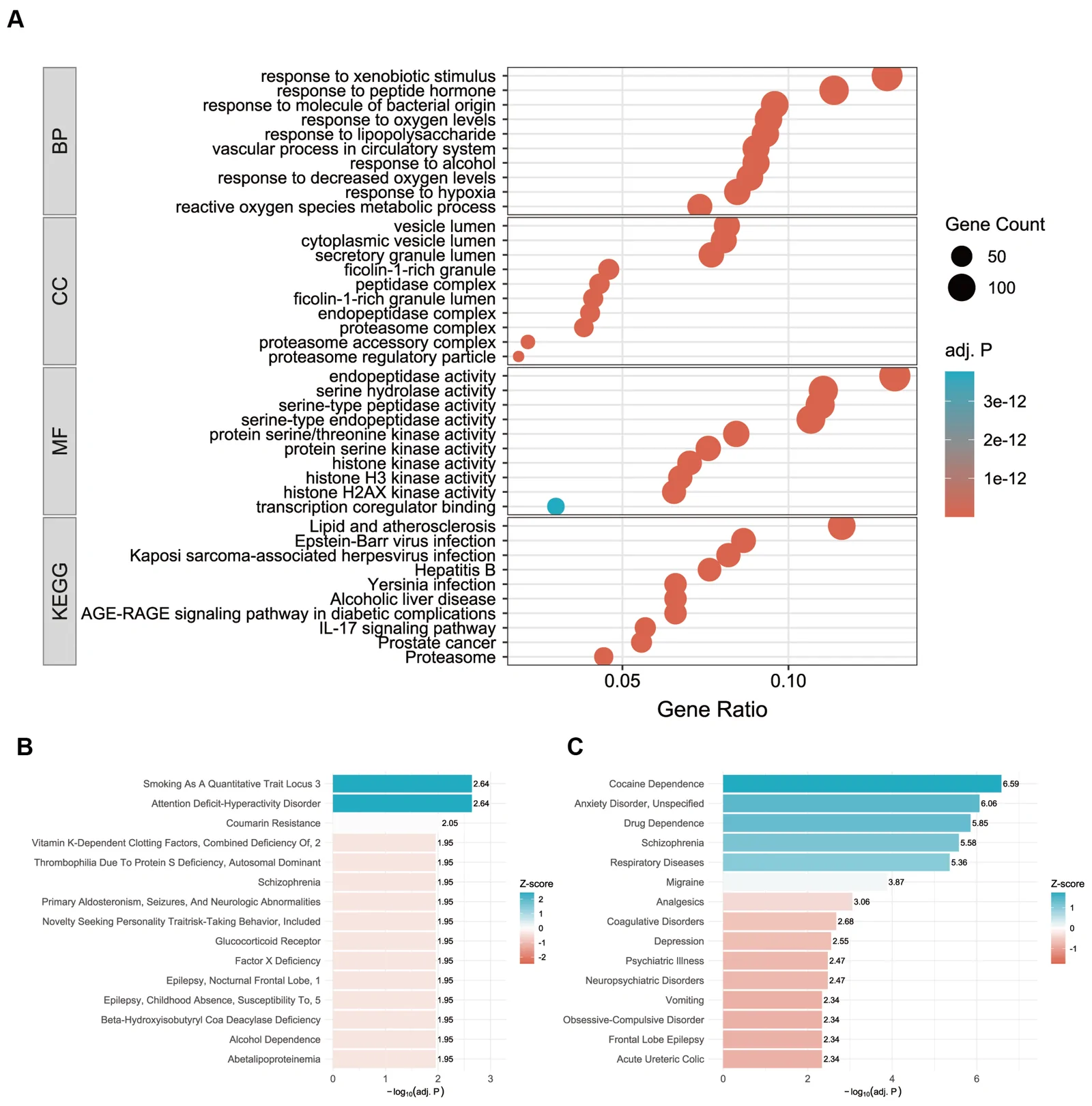
Visualization of enrichment patterns derived from putatively annotated compounds of NXLLS. (**A**) Bubble plots illustrating GO and KEGG enrichment patterns derived from putative target associations of 8 representative compounds annotated from LC–MS profiling of NXLLS. The top 10 enriched terms are ranked according to Gene Ratio. Dot size represents the number of associated genes (Gene Count), and color indicates the adjusted *p* value. BP = biological processes; CC = cellular components; MF = molecular functions. (**B**,**C**) Bar graphs showing disease enrichment analysis. OMIM (**B**) and TTD (**C**) enrichment plots were generated based on putative targets associated with 8 representative compounds annotated from LC–MS profiling of NXLLS. The top 15 enriched terms are ranked by Gene Ratio. Bar length indicates adjusted *p* value, and color represents the Z-score of the Gene Ratio.

Based on the 8 representative compounds supported by LC–MS annotation or HPLC authentic-standard confirmation, exploratory enrichment and network analyses were conducted to assess the potential biological relevance of NXLLS. GO biological process analysis indicated that the putative targets were primarily associated with responses to xenobiotic stimuli, oxygen levels and hypoxia, reactive oxygen species metabolism, and inflammatory stimuli (Figure 2A). GO cellular component analysis indicated enrichment in vesicle and secretory granule lumens, peptidase complexes, and proteasome-associated components. GO molecular function analysis identified enrichment in endopeptidase activity, serine-type peptidase activity, protein serine/threonine kinase activity, and histone kinase activity. KEGG analysis further identified the proteasome, IL-17, and AGE–RAGE signaling pathways, together with pathways associated with inflammatory and metabolic regulation. Some GO and KEGG terms involved biological processes relevant to cancer biology, including protein homeostasis, oxidative stress, hypoxia responses, inflammatory signaling, and kinase regulation. Disease enrichment analyses based on OMIM and TTD annotations showed that the predicted targets were primarily associated with neurobehavioral traits, substance dependence and neuropsychiatric disorders, respiratory diseases, and coagulation-related conditions (Figure 2B,C). These heterogeneous prediction-based associations were considered exploratory and hypothesis-generating and were not interpreted as evidence of a specific pharmacological mechanism.

### 3.3. NXLLS Suppresses Survival, Migration, and Invasion of Toledo DLBCL Cells

To evaluate the biological activity of NXLLS, we first compared the effects of NXLLS extracts prepared using different solvents on Toledo cell viability. NXLLS extracts prepared using EtOH, DMSO, HCl, or PBS all significantly inhibited Toledo cell viability relative to their corresponding solvent controls (Table 3). The calculated IC_50_ values ranged from 1.39 to 4.93 mg/mL for NXLLS extracts, whereas solvent controls exhibited substantially higher IC_50_ values (3.41–16.33 mg/mL), with the PBS control failing to reach 50% inhibition within the tested concentration range.

**Figure 3:**
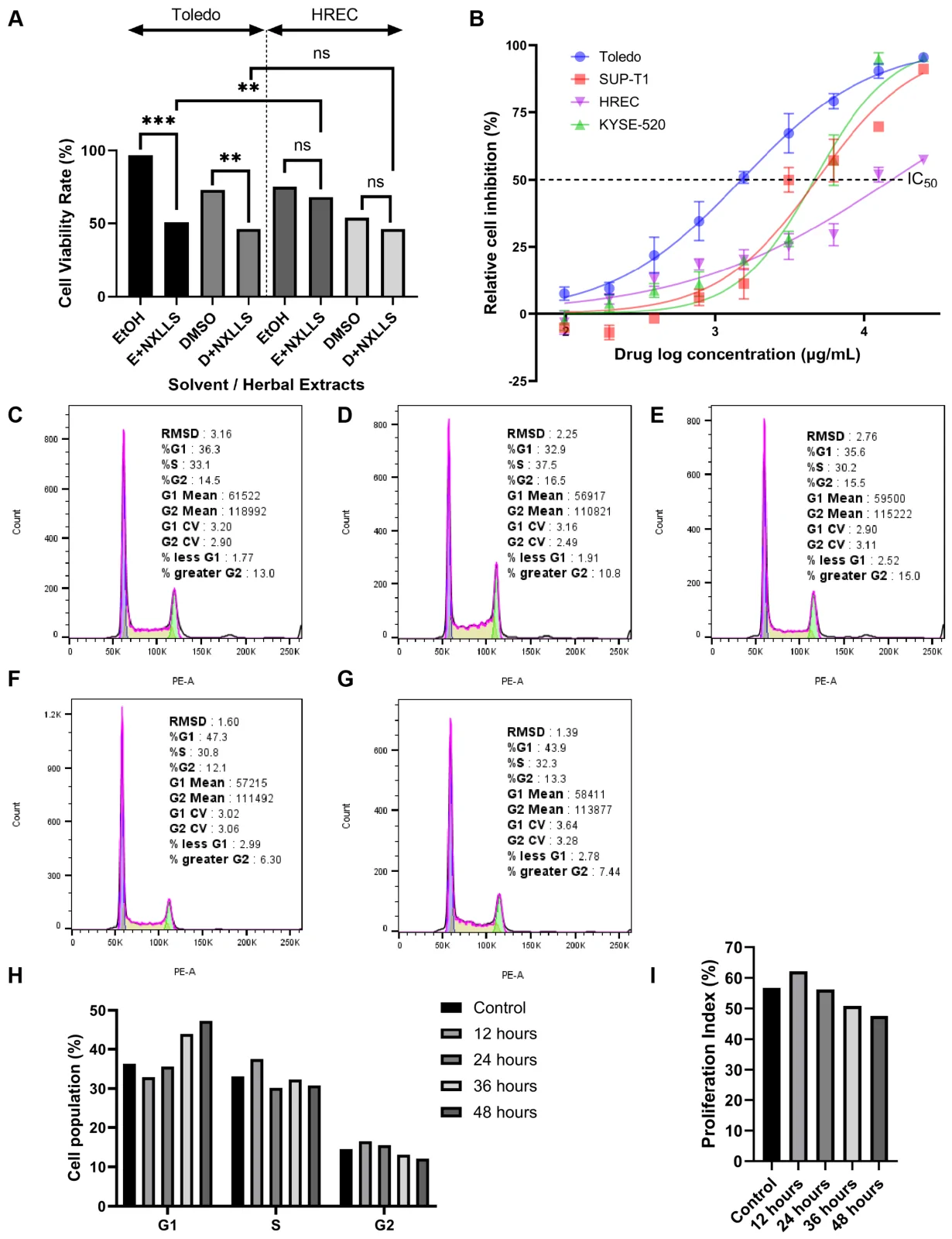
Cytotoxic effects of NXLLS on Toledo cells. (**A**) Comparative cytotoxic effects of EtOH- and DMSO-extracted NXLLS versus their respective vehicle controls in Toledo and HREC cells. (**B**) Comparison of cancer cell inhibition rates across different cell lines treated with NXLLS dissolved in EtOH. (**C**–**G**) Representative flow cytometry profiles showing the cell-cycle distributions of Toledo cells following NXLLS treatment. (**H**,**I**) Descriptive quantification of G_1_, S, and G_2_ phase populations (**H**) and the proliferation index (**I**) during NXLLS treatment. Statistical significance is indicated as ** *p <* 0.01, *** *p <* 0.001; ns = not significant. E+NXLLS = EtOH + NXLLS; D+NXLLS = DMSO + NXLLS.

Among the extraction methods, EtOH- and DMSO-extracted NXLLS exhibited the lowest IC_50_ values (1.51 and 1.39 mg/mL, respectively), while HCl- and PBS-extracted preparations were less effective. Two-way ANOVA further showed that treatment with EtOH- or DMSO-extracted NXLLS significantly reduced Toledo cell viability compared with the respective solvent control (Figure 3A). These results indicate that the observed effects were attributable to bioactive compounds present in NXLLS rather than the extraction solvents.

To explore whether the effects of NXLLS differed between Toledo and non-tumor cells, we also examined HREC cells. In HREC cells, neither EtOH- nor DMSO-extracted NXLLS significantly reduced cell viability compared with the corresponding vehicle control, indicating that the observed reduction in HREC viability could not be distinguished from the vehicle effect (Figure 3A). Under the tested conditions, EtOH-extracted NXLLS produced a greater reduction in viability in Toledo cells than in HREC cells (*p* < 0.01), whereas the effects of DMSO-extracted NXLLS did not differ significantly between the two cell lines. Based on its NXLLS-associated inhibitory effect in Toledo cells and the differential response observed between Toledo and HREC cells under the tested conditions, the EtOH extract was selected for subsequent experiments.

Next, we examined whether the inhibitory effects of NXLLS extend to other cancer types using representative SUP-T1 and KYSE-520 cell lines (Figure 3B). NXLLS reduced cell viability in a dose-dependent manner in all cell lines tested. However, marked differences in sensitivity were observed among cell types. Toledo cells were the most responsive to NXLLS, with an IC_50_ value of 1.51 ± 0.18 mg/mL, whereas substantially higher concentrations were required to achieve similar growth inhibition in SUP-T1 (4.84 ± 0.49 mg/mL), KYSE-520 (4.67 ± 0.36 mg/mL) cells (Table 4). The non-tumor HREC cells exhibited the lowest sensitivity, with an IC_50_ value of 15.23 ± 1.34 mg/mL, approximately 10-fold higher than that observed in Toledo cells. These results suggest that NXLLS exerts preferential inhibitory effects against Toledo cells while exhibiting comparatively limited toxicity toward non-tumor cells.

We next compared the viability-reducing activity of NXLLS with that of representative agents included in the CHOP chemotherapy regimen. Because NXLLS is a complex herbal formulation with no defined molecular weight, its IC_50_ values are reported on a mass basis (µg/mL). The IC_50_ values of the CHOP agents were initially determined from mass-based concentration data and subsequently converted to molar concentrations using their respective molecular weights; both units are presented in Table 5. In Toledo cells, the IC_50_ value of NXLLS was 1.51 ± 0.18 mg/mL, whereas the IC_50_ values of cyclophosphamide, doxorubicin, vincristine, and prednisone were 4.76 ± 0.56, 0.15 ± 0.03, 0.97 ± 0.17, and 16.80 ± 2.34 µg/mL, respectively. These comparisons are descriptive only, as direct mass-based comparisons between a complex herbal extract and purified chemotherapeutic agents have inherent limitations, particularly for agents such as cyclophosphamide that require metabolic activation. Within these limitations, the findings suggest that NXLLS treatment is associated with reduced viability in Toledo DLBCL cells under the in vitro conditions examined. Further studies using additional DLBCL models and in vivo systems are required before any therapeutic relevance can be assessed.

To gain insights into the molecular processes underlying the growth-inhibitory effects of NXLLS, we examined cell-cycle progression by flow cytometry. The proportion of Toledo cells in the G1 phase was higher than that of the control at the later time points, although the changes across the full time course were not monotonic (Figure 3C–H). The PI also showed a non-monotonic pattern, with an initial increase followed by a decrease at the later time points (Figure 3I). These descriptive observations suggest that NXLLS may alter cell-cycle distribution and potentially promote G1-phase accumulation at later time points.

**Figure 4:**
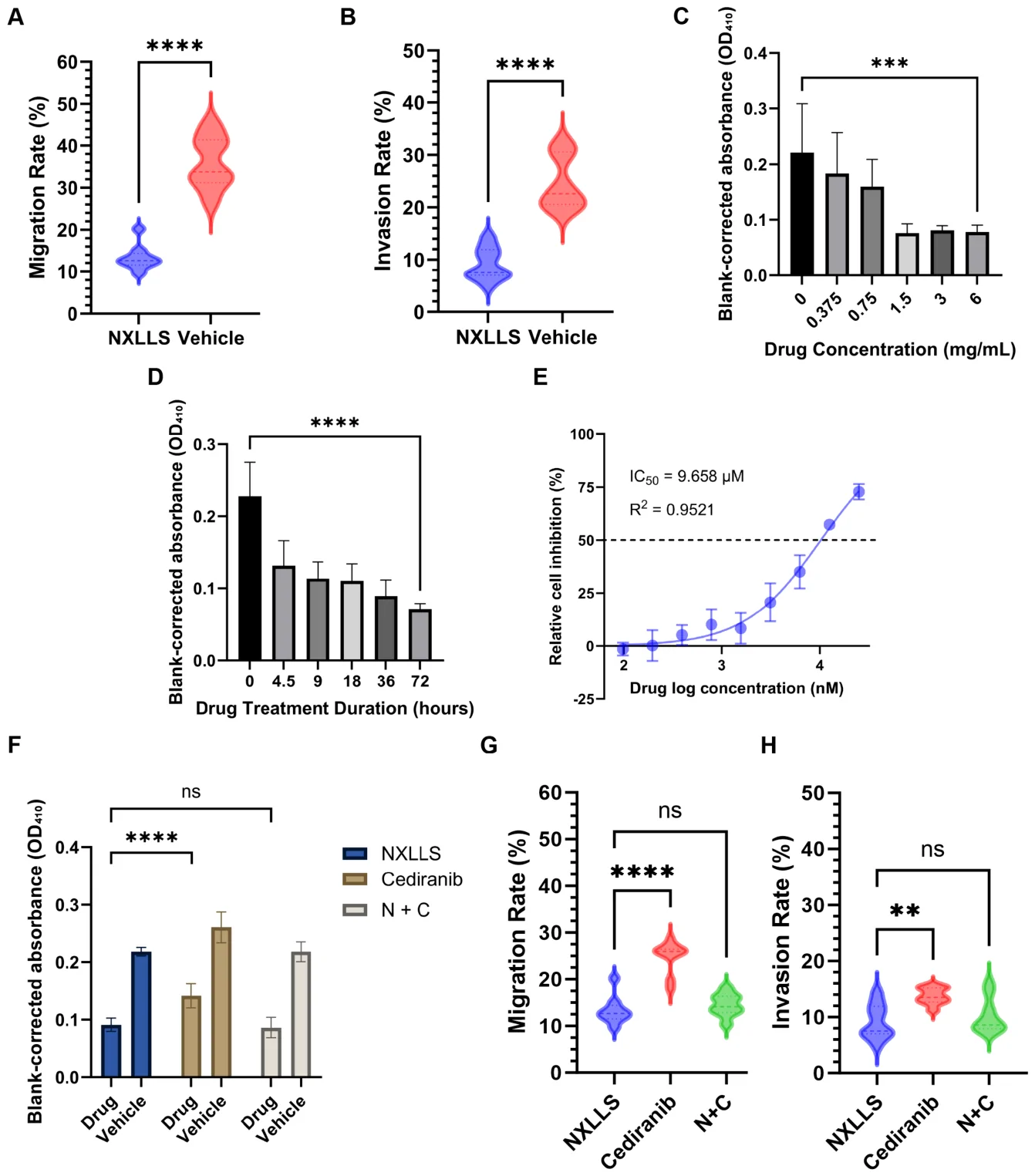
Effects of NXLLS on migration, invasion and VEGF-associated ELISA signal in Toledo Cells. (**A**,**B**) Transwell migration and invasion assays showing significant inhibition following NXLLS treatment. (**C**,**D**) Dose- and time-dependent decrease in VEGF-associated ELISA signal after NXLLS exposure. (**E**) Dose–response curve of the VEGF inhibitor cediranib. (**F**) Comparison of VEGF-associated ELISA signals following treatment with NXLLS, cediranib, or the combination. (**G**,**H**) Transwell migration and invasion assays comparing the effects of NXLLS, cediranib, and their combination on Toledo cells. Statistical significance is indicated as ** *p <* 0.01, *** *p <* 0.001, **** *p <* 0.0001; ns = not significant. N+C = NXLLS + cediranib.

In addition, we found that NXLLS treatment significantly decreased both the migratory and invasive capacities of Toledo cells (Figure 4A,B). Because VEGF is associated with tumor progression, migration, and invasion, we examined whether NXLLS treatment was accompanied by changes in the VEGF-associated signal in Toledo cell culture supernatants. The indirect ELISA signal decreased with increasing NXLLS concentration and treatment duration (Figure 4C,D). Cediranib, included as a pharmacological comparator, exhibited an estimated IC_50_ of 9.66 µM (Figure 4E). Co-treatment with NXLLS and cediranib did not further reduce the blank-corrected VEGF-associated ELISA signal (OD410) compared with NXLLS alone (Figure 4F). In the Transwell assays, migration and invasion were significantly lower following NXLLS treatment than following cediranib treatment under the tested conditions. Co-treatment with NXLLS and cediranib did not further reduce migration or invasion compared with NXLLS alone (Figure 4G,H). Collectively, these findings support a possible association between NXLLS treatment and altered VEGF-related signaling, although further studies are required to determine whether VEGF directly contributes to the observed effects.

### 3.4. RNA-seq Analysis Reveals Stress-Response Suppression and HDAC-Related Epigenetic Changes Following NXLLS Treatment

**Figure 5:**
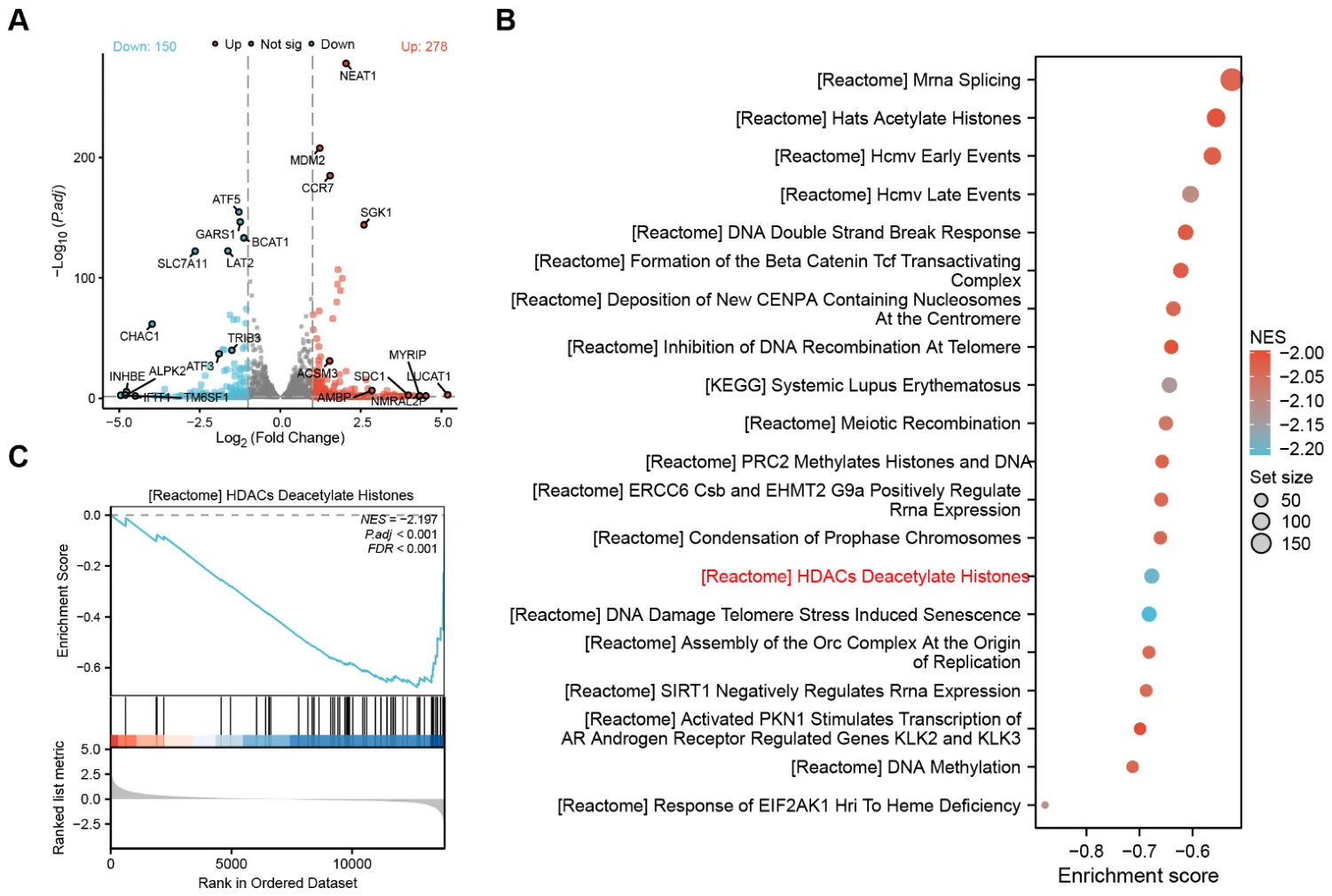
Differential gene expression and pathway analyses of NXLLS-treated Toledo cells. (**A**) Volcano plot showing significantly up-regulated (red) and down-regulated (blue) genes in NXLLS-treated Toledo cells. (**B**) GSEA bubble plot depicting gene sets that were negatively enriched following NXLLS treatment. Dot size represents gene set size, and normalized enrichment score (NES) reflects direction and magnitude of enrichment. (**C**) GSEA enrichment plot showing significant negative enrichment of the *HDACs deacetylate histones* pathway in NXLLS-treated Toledo cells.

To further investigate the potential molecular mechanisms of NXLLS, we analyzed RNA-seq data from Toledo cells treated with NXLLS. Differentially expressed genes (DEGs) were defined using an adjusted *p*-value < 0.05 and an absolute log2 fold change (|log2FC|) > 1. Based on these criteria, 428 DEGs were identified, including 278 upregulated and 150 downregulated genes (Figure 5A). Downregulated genes included *ATF3*, *ATF5*, *CHAC1*, and *SLC7A11*, which are implicated in oxidative stress responses, transcriptional regulation, and redox homeostasis. *TRIB3*, *BCAT1*, *LAT2*, and *INHBE*, which are associated with metabolic regulation and signal transduction, were also downregulated. In contrast, *NEAT1*, which has been reported to promote cancer cell survival and proliferation, was upregulated.

GSEA revealed significant negative enrichment of multiple pathways related to chromatin organization, DNA damage response, and immune processes in cells treated with NXLLS (Figure 5B). The complete GSEA results, including the enrichment score, normalized enrichment score, nominal *p* value, adjusted *p* value, and core enriched genes for all analyzed gene sets, are provided in Supplementary Document S6. Among these, the DNA damage/telomere stress–induced senescence pathway exhibited the strongest negative enrichment, consistent with coordinated downregulation of genes involved in this senescence-associated pathway. In parallel, several epigenetic regulatory pathways were significantly down-regulated, including PRC2 methylates histones and DNA, DNA methylation, and HDACs deacetylate histones, suggesting a coordinated impact of NXLLS on chromatin state and transcriptional regulation. Notably, the HDACs deacetylate histones pathway showed significant negative enrichment in NXLLS-treated Toledo cells (Figure 5C) (normalized enrichment score = −2.197; adjusted *p <* 0.001). The enrichment plot demonstrated that the genes involved in histone deacetylation were predominantly distributed toward the down-regulated end of the ranked gene list, consistent with coordinated transcriptional repression of HDAC-associated genes.

Altogether, these data suggest that NXLLS suppresses cellular stress-response pathways and HDAC-associated epigenetic programs, supporting a role for NXLLS in modulating chromatin regulation and stress-adaptive signaling in Toledo cells.

### 3.5. NXLLS Regulates Molecular Dynamics Associated with Cellular Stress

**Figure 6:**
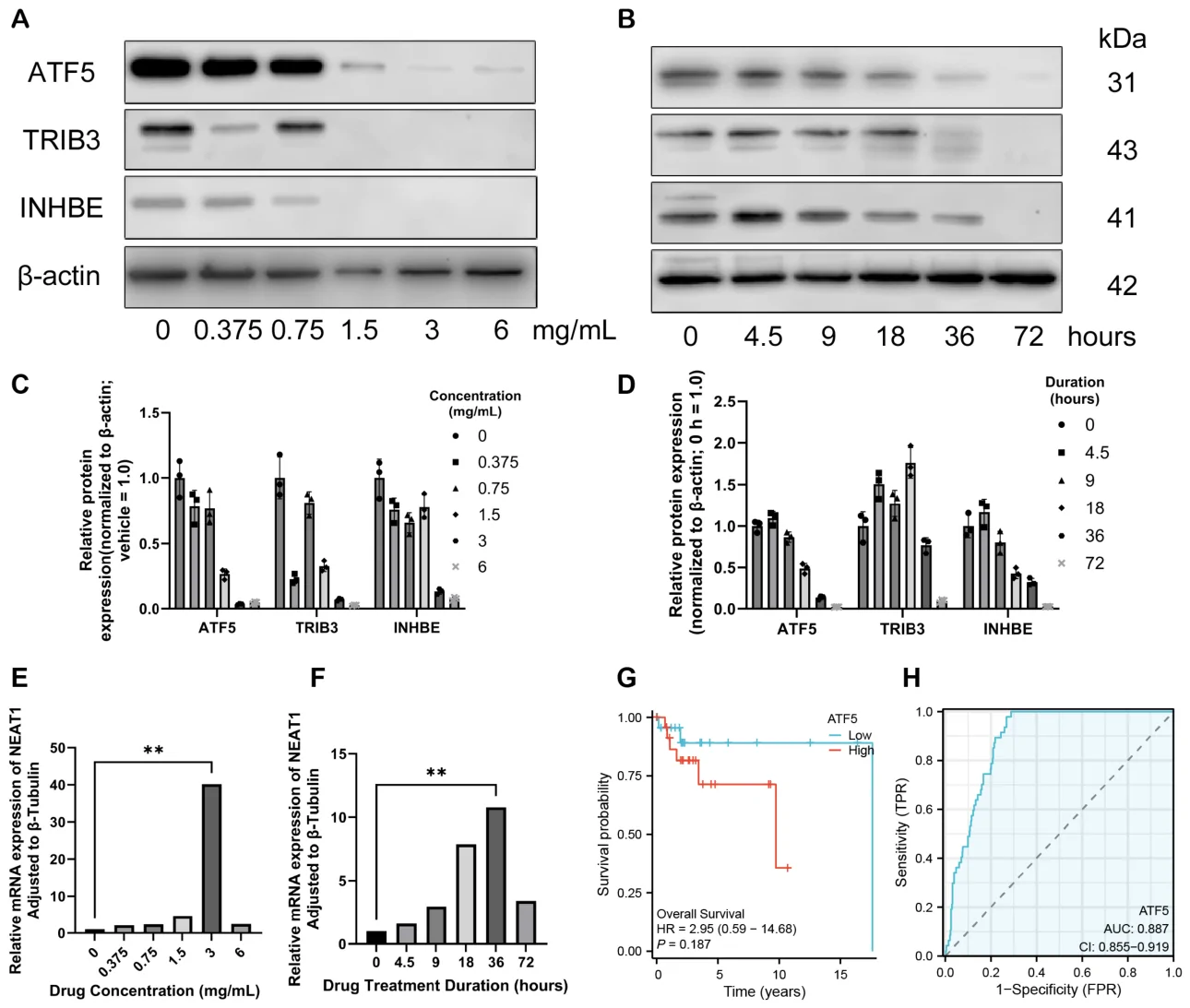
Evaluation of gene and protein expression of representative targets identified through transcriptomic analysis in Toledo cells. (**A**,**B**) Western blot analysis showing dose- and time-dependent regulation of ATF5, TRIB3, and INHBE following NXLLS treatment. (**C**,**D**) Densitometric quantification of the protein bands shown in (**A**,**B**), normalized to *β*-actin and expressed relative to the 0 mg/mL or 0 h control. The concentration- and time-course Western blot densitometry in (**C**,**D**) is presented descriptively without inferential statistical testing. (**E**,**F**) qPCR analysis showing changes in *NEAT1* expression levels, normalized to β-tubulin. (**G**) Kaplan–Meier survival analysis of *ATF5* using publicly available TCGA DLBCL datasets. (**H**) Exploratory receiver operating characteristic curve descriptively comparing *ATF5* expression between 47 TCGA-DLBC primary tumor samples and 444 GTEx reference samples, comprising 337 whole-blood samples and 107 EBV-transformed lymphocyte samples. The AUC and its 95% CI, estimated using DeLong’s method, are shown. Given the differences in tissue source and sample composition between the two groups, the results should be interpreted as exploratory and for reference only. AUC = area under the curve; CI = confidence interval; FPR = false-positive rate; HR = hazard ratio; TPR = true-positive rate. ** *p* < 0.01.

To further examine selected targets identified by RNA-seq following NXLLS treatment, ATF5, TRIB3, and INHBE were evaluated at the protein level, whereas *NEAT1* was assessed at the transcript level by qPCR. The protein abundance of ATF5, TRIB3, and INHBE decreased with increasing NXLLS concentration and treatment duration, consistent with the direction of their changes in the RNA-seq analysis (Figure 6A–D). Similarly, qPCR showed that *NEAT1* transcript levels increased following NXLLS treatment, reaching their highest levels at 3 mg/mL and after 36 h of treatment (Figure 6E,F). However, *NEAT1* expression decreased markedly at the highest concentration (6 mg/mL) and the longest treatment duration (72 h), suggesting that its induction may be transient and attenuated under prolonged or high-intensity NXLLS exposure. These findings indicate that *NEAT1* is dynamically regulated in response to NXLLS treatment and may participate in an adaptive cellular response.

We also examined the potential clinical associations of the selected genes using publicly available RNA-seq datasets from DLBCL patients in TCGA database. Kaplan–Meier survival analysis was performed to evaluate the potential prognostic relevance of *ATF5*, *TRIB3*, *INHBE*, and *NEAT1* expression in DLBCL patients. Among the 48 patients included in the analysis, 9 deaths were recorded. No statistically significant association between the expression of any of the four genes and overall survival was detected in the TCGA-DLBC cohort (Figure 6G, Supplementary Document S7). For *ATF5*, the estimated HR was 2.95 (95% CI, 0.59–14.68; *p* = 0.187). Given the small number of events and wide confidence intervals, these survival analyses were inconclusive. For an exploratory descriptive comparison, a ROC curve analysis was performed. Among the four candidates, *ATF5* exhibited the largest AUC in this cross-dataset comparison, with an area under the curve (AUC) of 0.887 (Figure 6H), followed by *TRIB3* (0.772), *INHBE* (0.669) and *NEAT1* (0.615) (Supplementary Document S7). The clinical significance of NXLLS- associated molecular changes remains to be further investigated.

### 3.6. HDAC Isoforms and Histone Acetylation Are Dynamically Regulated by NXLLS

**Figure 7:**
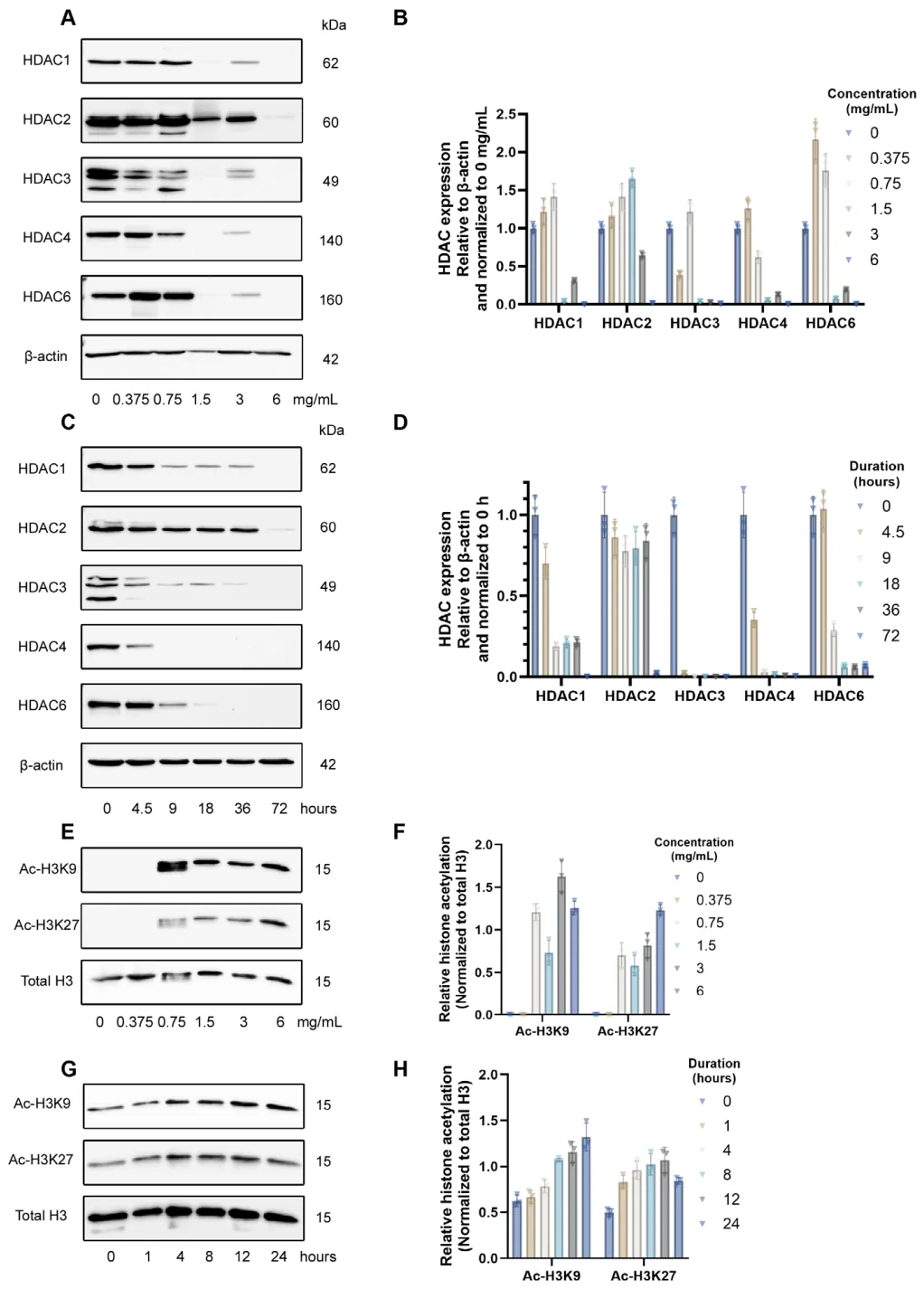
Effects of NXLLS on HDAC expression and histone acetylation in Toledo cells. (**A**) Western blot analysis showing dose-dependent changes in HDAC isoform expression following NXLLS treatment. (**B**) Densitometric quantification of HDAC protein expression shown in (**A**), normalized to β-actin and expressed relative to the 0 mg/mL control. (**C**) Western blot analysis showing time-dependent changes in HDAC isoform expression following NXLLS treatment. (**D**) Densitometric quantification of HDAC protein expression shown in (**C**), normalized to β-actin and expressed relative to the 0 h control. (**E**,**G**) Western blot analysis showing dose- and time-dependent alterations in histone H3 acetylation marks (Ac-H3K9 and Ac-H3K27) following NXLLS treatment. (**F**,**H**) Densitometric quantification of histone acetylation levels shown in (**E**,**G**), normalized to total H3. Data are presented as the mean ± SD of three biological replicates, with individual data points shown. The concentration- and time-course densitometric data in (**B**,**D**,**F**,**H**) are presented descriptively without inferential statistical testing.

Our GSEA revealed significant negative enrichment of HDAC-associated pathways in NXLLS-treated Toledo cells, consistent with previous studies showing a crucial role for HDAC-associated epigenetic regulation in DLBCL [29,30]. To further investigate this observation, we examined the expression of representative HDAC family members and histone acetylation status. Our Western blot analysis showed concentration- and time-associated changes in the expression of multiple HDAC isoforms, including the Class I HDACs (HDAC1, HDAC2, and HDAC3) and the Class II HDACs (HDAC4 and HDAC6) (Figure 7A–D). Reductions at higher concentrations and longer treatment durations were particularly pronounced for HDAC3, HDAC4, and HDAC6, whose expression was nearly abolished under these conditions. HDAC1 showed a more gradual decline, whereas HDAC2 exhibited a non-monotonic concentration response, with an apparent increase at 0.75 mg/mL followed by a reduction at higher concentrations. These patterns suggest differential responses among HDAC family members following NXLLS exposure. We also found that NXLLS increased the levels of the histone acetylation markers, Ac-H3K9 and Ac-H3K27 (Figure 7E–H). The histone acetylation results should be interpreted as semiquantitative because the signals in the untreated group were close to background. The induction of histone acetylation was most evident at intermediate concentrations and treatment durations, whereas a modest reduction was observed at the highest concentration and latest time point, possibly reflecting secondary cellular responses associated with prolonged exposure. To assess the clinical relevance of the HDAC isoforms affected by NXLLS, we analyzed TCGA DLBCL datasets. None of the HDAC genes examined (HDAC1, HDAC2, HDAC3, HDAC4, or HDAC6) showed a significant association with overall survival. Nevertheless, the reductions observed in several HDAC isoforms, coupled with increased H3K9 and H3K27 acetylation, suggest that NXLLS treatment is associated with broad epigenetic changes in Toledo DLBCL cells.

### 3.7. NXLLS Induces Coordinated Repression of ATF5-Related Cellular Stress and HDAC-Associated Epigenetic Programs

**Figure 8:**
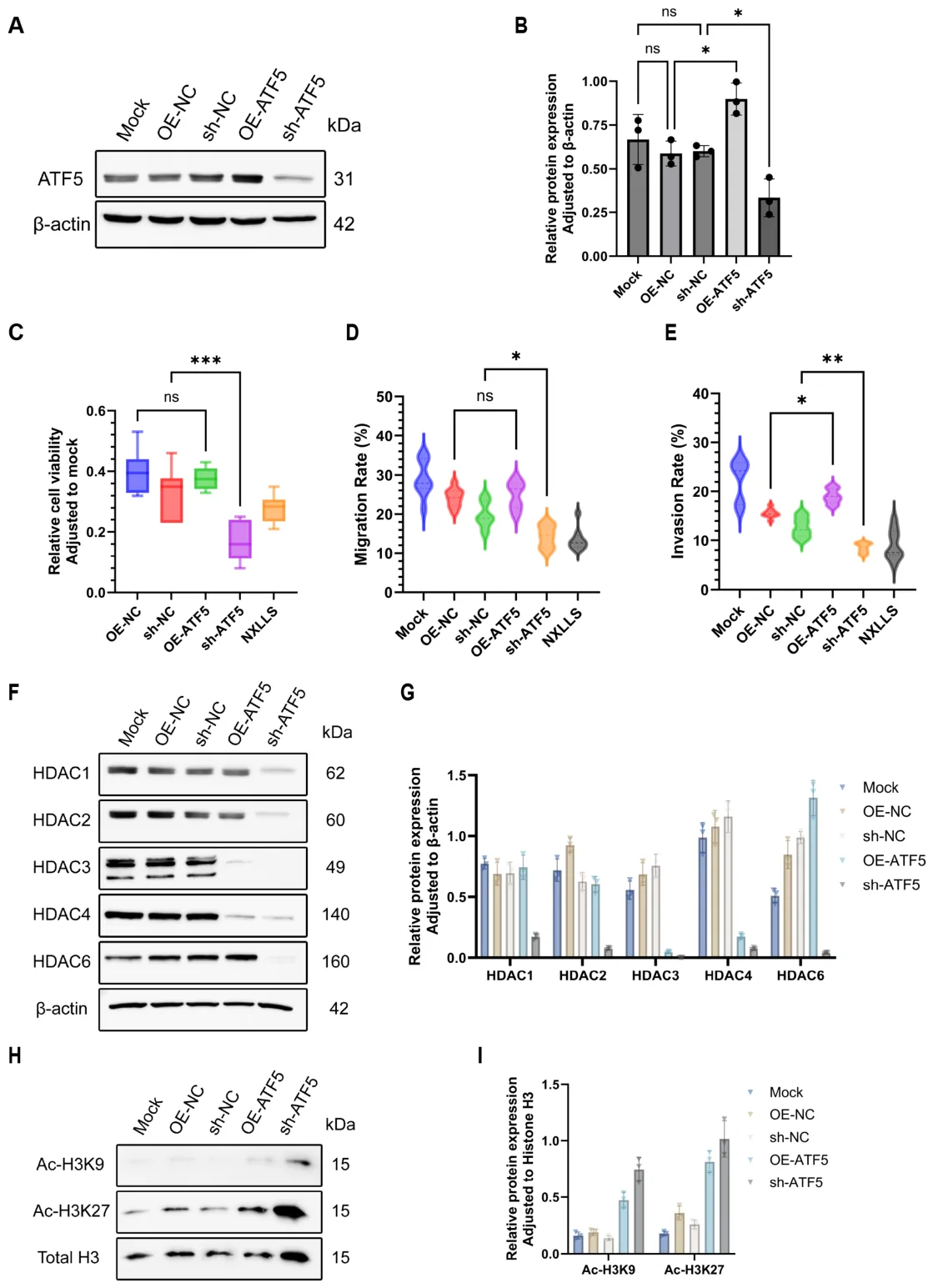
Functional characterization of *ATF5*-overexpressing and *ATF5*-knockdown Toledo cells. (**A**,**B**) Western blot analysis confirming *ATF5* overexpression and knockdown efficiency. (**C**) Cell viability analysis of ATF5-modified cells and NXLLS-treated cells. (**D**,**E**) Transwell migration and invasion assays of ATF5-modified cells and NXLLS-treated cells. (**F**) Western blot analysis of HDAC family member expression in ATF5-modified cells. (**G**) Densitometric quantification of HDAC protein expression shown in (**F**), normalized to β-actin. (**H**) Western blot analysis of Ac-H3K9, and Ac-H3K27 expression in ATF5-modified cells. (**I**) Densitometric quantification of histone acetylation levels shown in (**H**), normalized to total H3. Statistical significance is indicated as * *p* < 0.05, ** *p* < 0.01, *** *p* < 0.001; ns = not significant.

Based on our transcriptomic and TCGA data analyses, we explored the role of ATF5 in mediating the cellular response to NXLLS. Stable Toledo cell lines with *ATF5* overexpression or knockdown were generated and confirmed by Western blot analysis (Figure 8A,B). ATF5 protein expression in the OE-ATF5 group was approximately 1.8-fold higher than that in the OE-NC group. Functional characterization revealed that *ATF5* depletion markedly impaired cell viability compared with the control group (*p <* 0.001), whereas the modest *ATF5* overexpression achieved under the present experimental conditions was not associated with a significant change in viability (Figure 8C). We also observed that *ATF5* knockdown significantly reduced cell migration and invasion compared with the control (*p <* 0.05 and *p* < 0.01, respectively), whereas modest *ATF5* overexpression was not associated with a significant change in migration and produced only a modest increase in invasive capacity (Figure 8D,E). We also found that *ATF5* modulation contributes to the HDAC expression. *ATF5* knockdown resulted in a marked reduction in multiple HDAC isoforms, including HDAC1, HDAC2, HDAC3, HDAC4, and HDAC6 (Figure 8F,G). Modest *ATF5* overexpression was associated with reduced HDAC3 and HDAC4 protein levels, with minimal changes in HDAC1, HDAC2, and HDAC6 relative to controls. Concomitantly, *ATF5* knockdown showed increased levels of Ac-H3K9 and Ac-H3K27 (Figure 8H,I), showing a pattern similar to that observed following NXLLS treatment, whereas the modest *ATF5* overexpression produced more limited changes.

Taken together, these findings suggest that ATF5 contributes to the regulation of both DLBCL cell fitness and HDAC-associated epigenetic programs.

## 4. Discussion

In this study, we established the chemical profiles of NXLLS and identified putatively annotated compounds associated with cancer-related molecular pathway and immune regulation. A critical challenge in the study of multi-component herbal formulations is ensuring batch-to-batch consistency and establishing a reliable chemical basis. Our comprehensive profiling approach, integrating with TLC, HPLC, and LC–MS, provided a preliminary assessment of the compositional similarity of NXLLS samples, resulting in the selection of 8 representative compounds, including compounds confirmed using authentic standards and putatively annotated compounds, for downstream analyses. Our findings also demonstrate the underlying molecular mechanisms of NXLLS in Toledo DLBCL cells, which involve cellular stress and epigenetic reprogramming, including the role of ATF5 and the HDAC pathways.

NXLLS treatment was associated with reduced viability, migration, and invasion in Toledo DLBCL cells, accompanied by a decrease in the VEGF-associated ELISA signal. These findings suggest a possible association between NXLLS treatment and altered VEGF-related signaling, although reduced cell viability may have contributed to the observed decrease in the ELISA signal. Cediranib also affected cell viability, migration, and invasion; however, its combination with NXLLS did not produce additional inhibitory effects compared with NXLLS alone. Given the broad role of VEGF in tumor progression and clinical outcomes [31,32], further studies are needed to determine whether VEGF directly contributes to the response to NXLLS. The broader biological effects of NXLLS on Toledo DLBCL cells may reflect the collective actions of multiple natural constituents and signaling pathways.

To obtain a more comprehensive understanding of the molecular pathways involved, we leveraged RNA-seq transcriptomic data and our analysis revealed not only alterations in individual molecular targets, but also broader regulatory perturbations, including negative enrichment of gene sets related to chromatin-modifying processes such as HDAC-associated deacetylation, PRC2-mediated methylation, and DNA methylation. These observations are consistent with the well-established role of epigenetic dysregulation in the pathogenesis of DLBCL [33]. Among these pathways, HDAC-associated deacetylation represents a central mechanism of epigenetic regulation, in which histone deacetylases remove acetyl groups from lysine residues on histone tails, leading to chromatin condensation and transcriptional repression [34].

HDACs are key epigenetic regulators that control chromatin structure and gene expression by removing acetyl groups from histone and non-histone proteins [34]. Dysregulation of HDAC activity has been implicated in the pathogenesis of multiple cancers, including DLBCL, where complexes such as BCL6–HDAC3 mediate oncogenic transcriptional repression [35]. Aberrant upregulation of HDAC activity also contributes to the silencing of tumor suppressor genes [36,37]. Consequently, dysregulated HDAC-associated epigenetic regulation has been recognized as a therapeutically actionable vulnerability and has become the focus of ongoing efforts to develop targeted epigenetic therapies [36,38]. Importantly, the functional diversity of the HDAC isoforms reflects the molecular heterogeneity of DLBCL. For instance, Class I HDACs, including HDAC1, HDAC2, and HDAC3, play an essential role in lymphoma cell survival and are frequently upregulated in the ABC subtype, which is associated with a poor prognosis. Their elevated expression correlates with increased proliferation and resistance to apoptosis [7,39]. In contrast, class II HDACs, such as HDAC4 and HDAC6, exert distinct but critical functions. HDAC4 regulates B-cell differentiation by repressing transcription factors such as MEF2, and its dysregulation contributes to lymphomagenesis [33]. HDAC6, predominantly localized in the cytoplasm, modulates non-histone substrates such as *α*-tubulin, thus promoting cell survival and protein homeostasis, particularly in the ABC subtype, making it a promising therapeutic target [40,41]. The downstream consequences of aberrant HDAC activity are reflected in altered histone acetylation patterns. Ac-H3K9 and Ac-H3K27 represent the hallmark epigenetic marks of active promoters and enhancers [42]. A key oncogenic mechanism in DLBCL involves loss-of-function mutations in the histone acetyltransferases CREBBP and EP300, which are particularly prevalent in the GCB subtype. These alterations lead to global reductions in histone acetylation, including H3K27, resulting in widespread transcriptional repression and disease progression [43].

Collectively, the disruption of the dynamic balance between HDACs and histone acetyltransferases (HATs)—whether through HDAC overexpression or loss of HAT function—represents a fundamental epigenetic abnormality in DLBCL, with profound effects on its biological behavior and clinical outcome [34,36]. Consistent with these mechanistic frameworks, our data showed concentration- and time-associated changes in the expression of HDAC1, HDAC2, HDAC3, HDAC4, and HDAC6, with the patterns varying among individual isoforms, accompanied by increased levels of Ac-H3K9 and Ac-H3K27. These findings suggest that NXLLS-associated alterations in HDAC expression and histone acetylation occurred alongside growth inhibition and changes in cell-cycle distribution [44], although a causal relationship cannot be established from the present data. Together with the transcriptomic results, these data suggest a potential involvement of HDAC-related epigenetic modulation in the cellular response to NXLLS.

Beyond epigenetic dysregulation, aggressive DLBCL is driven by multiple stress-response and survival pathways. ATF5 is a stress-responsive transcription factor that is overexpressed in lymphoma, where it promotes cell survival under stress of the endoplasmic reticulum (ER) and confers resistance to apoptosis [45]. TRIB3, a stress-inducible pseudokinase, facilitates MYC-driven lymphomagenesis by stabilizing MYC and preventing its ubiquitin-mediated degradation [46,47]. INHBE, a member of the TGF-*β* superfamily, has been implicated in tumor growth and immune modulation in several malignancies, including hepatocellular and renal cell carcinoma [48,49]. *NEAT1*, a well-characterized oncogenic lncRNA, is frequently upregulated in DLBCL and promotes proliferation and invasion by sponging tumor suppressor miRNAs such as miR-495-3p [50]. Despite the significant expression changes in these genes induced by NXLLS, our TCGA data analysis indicates that the clinical relevance of most of them in DLBCL remains unclear. Exploratory analysis of *ATF5* indicated potential clinical relevance, although the limited cohort size precludes definitive conclusions regarding its diagnostic or prognostic value. Our findings further identify ATF5 as a potential downstream mediator of the cellular response to NXLLS, as *ATF5* manipulation recapitulated several cellular and epigenetic changes associated with NXLLS treatment. Particularly, *ATF5* overexpression and knockdown produced distinct changes in HDAC isoform expression and histone acetylation, suggesting a complex association between ATF5 and HDAC-related epigenetic regulation. However, the causal relationship between ATF5 and the effects of NXLLS remains to be established. Further studies are required to define the mechanistic role and clinical relevance of ATF5 in DLBCL.

This study has several limitations that should be acknowledged. First, all experiments were conducted in vitro. The non-tumor comparison was limited to HREC cells because suitable commercially available non-malignant B-cell or lymphoid cell models were not readily accessible, and primary human lymphoid cells could not be obtained within the scope of this study. Moreover, the effects observed in HREC cells at high extract concentrations could not be distinguished from the corresponding ethanol vehicle effect. Therefore, the comparison between Toledo and HREC cells does not establish DLBCL-specific selectivity. The lack of in vivo validation further limits conclusions regarding safety and clinical efficacy. Second, the bioactive constituents responsible for the observed effects of NXLLS have not been fully isolated or functionally validated, and their precise material basis remains to be elucidated. Activity-guided fractionation and targeted validation will be required to identify key bioactive components and clarify their individual contributions. Third, although both *ATF5* overexpression and knockdown were functionally investigated, the relatively modest *ATF5* overexpression achieved in this study (approximately 1.8-fold relative to OE-NC) may have limited the ability to detect phenotypic and molecular effects in the overexpression experiments. In addition, a successful rescue experiment involving *ATF5* overexpression in NXLLS-treated cells could not be achieved under the current experimental conditions. Therefore, the present findings support ATF5 as a potential downstream mediator but do not establish a definitive causal role in the NXLLS response. In addition, although viable cells were enriched and equal numbers of viable cells were seeded for the migration and invasion assays following *ATF5* manipulation, cell viability was not independently assessed during the assay period. Therefore, the observed changes in migration and invasion cannot be fully distinguished from potential secondary effects associated with altered cell survival. Furthermore, the observed changes in HDAC expression and histone acetylation remain associative and do not establish a causal role for HDAC modulation in the biological effects of NXLLS. At higher NXLLS concentrations, cytotoxicity may have contributed to the observed changes in HDAC proteins, histone acetylation, and VEGF-associated ELISA signals. Finally, the relatively small TCGA-DLBC cohort (*n* = 48) limits conclusions regarding the clinical relevance of *ATF5*, and validation in larger independent cohorts is warranted.

Future studies should extend these findings by examining additional stress-response and cell-death pathways and validating the effects of NXLLS in vivo. Although ATF4 was not significantly altered at the transcript level in the present RNA-seq dataset, its activity is substantially regulated at the translational level. Direct assessment of ATF4 protein, phosphorylated and total eIF2α, and other components of the integrated stress response will therefore be required to determine whether this pathway contributes to the cellular response to NXLLS. In addition, the observed downregulation of SLC7A11 raises the possibility that ferroptosis-related processes may be involved. This possibility should be evaluated using lipid peroxidation measurements, ferroptosis-associated molecular markers, and rescue experiments with ferrostatin-1 or liproxstatin-1. Finally, appropriate in vivo DLBCL models will be needed to assess antitumor activity, toxicity, pharmacological exposure, and the relevance of the proposed ATF5-, HDAC-, ISR-, and ferroptosis-associated changes under physiologically relevant conditions.

## 5. Conclusions

NXLLS treatment reduced cell viability, inhibited migration and invasion, and was associated with altered cell-cycle distribution and potential G1-phase accumulation at later time points in Toledo DLBCL cells. Integrated analyses identified potential molecular targets associated with the cellular response to NXLLS, among which ATF5 was further functionally investigated. *ATF5* knockdown largely recapitulated the cellular effects observed following NXLLS treatment, supporting ATF5 as a potential downstream mediator of the NXLLS response. These findings, together with the observed alterations in HDAC expression and histone acetylation, suggest a potential association between ATF5- and HDAC-related epigenetic modulation in the cellular response to NXLLS. Overall, this study identifies ATF5- and HDAC-associated epigenetic changes as molecular features associated with the response to NXLLS in Toledo DLBCL cells and provides a basis for further investigation of their mechanistic relationships.

## Figures and Tables

**Table 1:** Details of Medicinal Materials in NXLLS.

Pinyin Name	Scientific Name	Part Used	w/w (%)
Xiakucao	*Prunella vulgaris* L. (Lamiaceae)	Fruiting spike	25
Mulike	*Magallana gigas* (Thunberg, 1793) (Ostreidae)	Calcined shell	10
Zhebeimu	*Fritillaria thunbergii* Miq. (Liliaceae)	Bulb	10
Tianhuafen	*Trichosanthes kirilowii* Maxim. (Cucurbitaceae)	Root	10
Xuanshen	*Scrophularia ningpoensis* Hemsl. (Scrophulariaceae)	Root	15
Jiegeng	*Platycodon grandiflorus* (Jacq.) A.DC. (Campanulaceae)	Root	10
Baizhi	*Angelica dahurica* (Hoffm.) Benth. & Hook.f. exFranch. & Sav. (Apiaceae)	Root	10
Shudahuang	*Rheum palmatum* L. (Polygonaceae)	Root and rhizome (processed)	10

**Table 2:** Representative compounds supported by LC–MS putative annotation or HPLC authentic-standard confirmation in NXLLS.

Compound	RT (min)	Observed m/z	Ionisation Mode	Adduct	Molecular Formula	Mass Error (ppm)	PubChem CID	Identification Level
(+)-Catechin	16.24	291.08642	Positive	[M + H]^+^	C15H14O6	0.36	9064	Putatively annotated by LC–MS
Sennoside B	21.09	861.18826	Negative	[M − H]^−^	C42H38O20	−0.12	91440	Putatively annotated by LC–MS
Rosmarinic acid	19.97	359.07732	Negative	[M − H]^−^	C18H16O8	0.22	5281792	Putatively annotated by LC–MS
Imperatorin	38.51	271.09634	Positive	[M + H]^+^	C16H14O4	−0.54	10212	Putatively annotated by LC–MS
Quercetin	22.94	301.03542	Negative	[M − H]^−^	C15H10O7	0.15	5280343	Confirmed by authentic-standard comparison and HPLC co-injection
Kaempferol	24.85	285.04066	Negative	[M − H]^−^	C15H10O6	0.7	5280863	Confirmed by authentic-standard comparison and HPLC co-injection
Osthole	30.29	245.11717	Positive	[M + H]^+^	C15H16O3	−0.21	10228	Putatively annotated by LC–MS
Rutin	19.81	609.14728	Negative	[M − H]^−^	C27H30O16	1.92	5280805	Confirmed by authentic-standard comparison and HPLC co-injection

**Table 3:** IC_50_ Values of NXLLS Extracts Prepared Using Different Solvents in the Toledo Cells.

Extraction Solvent	NXLLS Extract IC_50_ (mg/mL)	R^2^	Solvent Control IC_50_ (mg/mL)	R^2^
EtOH	1.51	0.9749	8.74	0.9411
DMSO	1.39	0.9876	3.41	0.9623
HCl	3.36	0.9565	16.33	0.9173
PBS	4.93	0.9634	Not reached	N/A

**Table 4:** IC_50_ Values of NXLLS in Different Cell Lines.

Cell Line	Mass (mg/mL)	R^2^
Toledo	1.51 ± 0.18	0.9856
SUP-T1	4.84 ± 0.49	0.9511
KYSE-520	4.67 ± 0.36	0.9632
HREC	15.23 ± 1.34	0.9278

**Table 5:** IC_50_ Values of Representative Chemotherapy Agents in Toledo Cells.

Chemotherapy Agent	Molar (µM)	Mass (µg/mL)
Cyclophosphamide	18.22 ± 2.14	4.76 ± 0.56
Doxorubicin	0.27 ± 0.05	0.15 ± 0.03
Vincristine	1.05 ± 0.19	0.97 ± 0.17
Prednisone	46.87 ± 6.52	16.80 ± 2.34

## Data Availability

The RNA-sequencing data generated in this study have been deposited in the NCBI Gene Expression Omnibus (GEO) under accession number GSE346534. The other data presented in this study are available from the corresponding author upon reasonable request.

## Supplementary Materials

The following supporting information can be downloaded at https://www.mdpi.com/article/10.3390/biomedicines14092052/s1. Supplementary Document S1: Generation of stable ATF5-overexpression and knockdown Toledo cell lines. Supplementary Document S2: Quality control reports for NXLLS herbal materials. Supplementary Document S3: TLC identification. Supplementary Document S4: HPLC fingerprint similarity analysis of NXLLS batches. Supplementary Document S5: LC–MS chemical fingerprinting used to verify the phytochemical composition of NXLLS. Supplementary Document S6: Complete GSEA Results. Supplementary Document S7: Kaplan–Meier survival and ROC curve analyses of *TRIB3*, *INHBE*, *NEAT1*. Table S1: List of antibodies used in Western blotting, including catalog numbers, suppliers, and working dilutions. Figure S1: Regression model used to estimate the number of migrated or invaded cells.

## Abbreviations

The following abbreviations are used in this manuscript:

Ac-H3K9	Acetylated histone H3 lysine 9
Ac-H3K27	Acetylated histone H3 lysine 27
AUC	Area under the curve
CHOP	Cyclophosphamide, doxorubicin, vincristine, and prednisone
DEGs	Differentially expressed genes
DLBCL	Diffuse large B-cell lymphoma
ER	Endoplasmic reticulum
ELISA	Enzyme-linked immunosorbent assay
EtOH	Ethanol
HREC	Human renal epithelial cells
FPR	False-positive rate
GO	Gene ontology
GSEA	Gene set enrichment analysis
GTEx	Genotype-Tissue Expression
HDAC	Histone deacetylase
HPLC	High-performance liquid chromatography
HR	Hazard ratio
IC_50_	Half maximal inhibitory concentration
ISR	Integrated stress response
KEGG	Kyoto Encyclopedia of Genes and Genomes
LC–MS	Liquid chromatography–mass spectrometry
MOI	Multiplicity of infection
NXLLS	Nei-Xiao-Luo-Li-San
OMIM	Online Mendelian Inheritance in Man
PI	Proliferation index
PBS	Phosphate-buffered saline
RRID	Research Resource Identifier
ROC	Receiver operating characteristic
shRNA	Short hairpin RNA
TCM	Traditional Chinese medicine
TCGA	The Cancer Genome Atlas
TLC	Thin-layer chromatography
TPR	True-positive rate
TTD	Therapeutic Target Database
VEGF	Vascular endothelial growth factor
WST-1	Water-soluble tetrazolium salt-1
