# Supplementary material for: Responses to Nei-Xiao-Luo-Li-San in Toledo Diffuse Large B-Cell Lymphoma Cells: Potential Involvement of ATF5- and HDAC-Associated Epigenetic Changes
Source: Biomedicines. 2026 Sep 12;14(9):2052. doi: 10.3390/biomedicines14092052 (PMC13604743; doi:10.3390/biomedicines14092052)
Supplement: Supplementary file 1 [file biomedicines-14-02052-s001.zip › Supplementary Document S7 Kaplan–Meier survival and ROC curve analyses of TRIB3, INHBE, NEAT1.pdf]

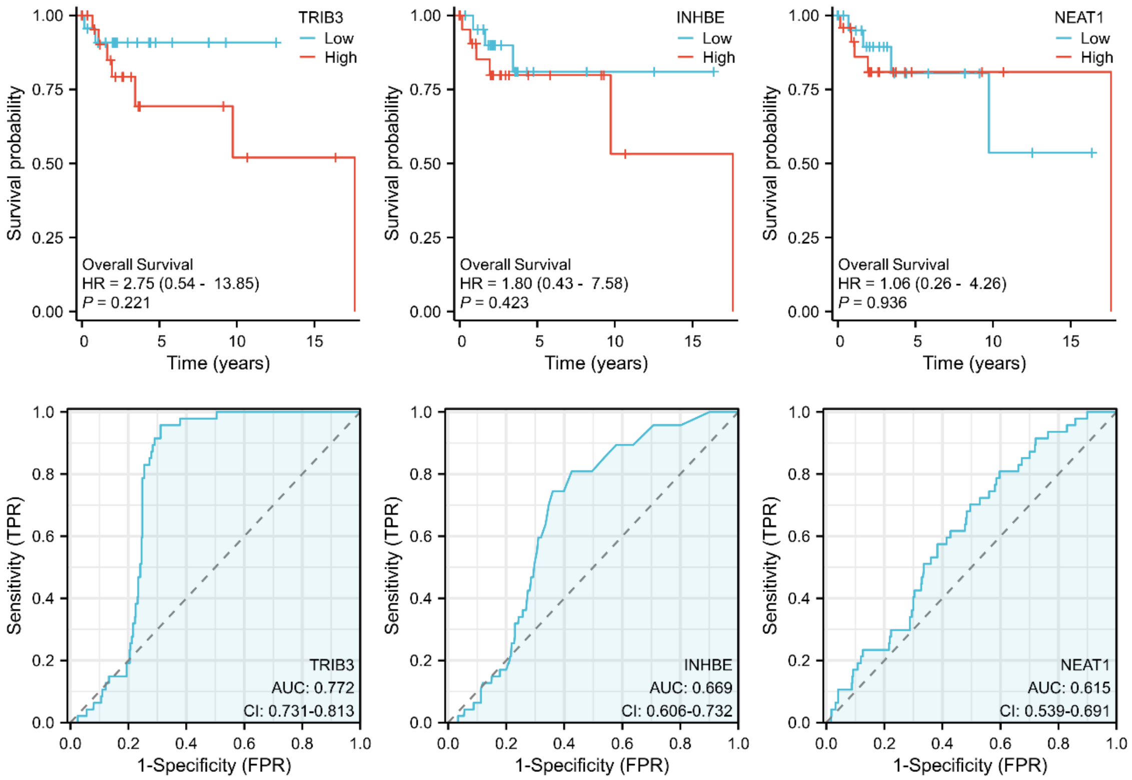

TRIB3
Low
High
Survival probability
1.00
0.75
0.50
0.25
0.00
Overall Survival
HR = 2.75 (0.54 - 13.85)
P = 0.221
0
5
10
15
Time (years)
INHBE
Low
High
Overall Survival
HR = 1.80 (0.43 - 7.58)
P = 0.423
NEAT1
Low
High
Overall Survival
HR = 1.06 (0.26 - 4.26)
P = 0.936
Sensitivity (TPR)
1-Specificity (FPR)
0.0
0.2
0.4
0.6
0.8
1.0
TRIB3
AUC: 0.772
CI: 0.731-0.813
INHBE
AUC: 0.669
CI: 0.606-0.732
NEAT1
AUC: 0.615
CI: 0.539-0.691
