# Supplementary material for: Responses to Nei-Xiao-Luo-Li-San in Toledo Diffuse Large B-Cell Lymphoma Cells: Potential Involvement of ATF5- and HDAC-Associated Epigenetic Changes
Source: Biomedicines. 2026 Sep 12;14(9):2052. doi: 10.3390/biomedicines14092052 (PMC13604743; doi:10.3390/biomedicines14092052)
Supplement: Supplementary file 1 [file biomedicines-14-02052-s001.zip › Supplementary Table S1.pdf]

Supplementary Table S1.

List of antibodies used in Western blotting, including catalog numbers, suppliers, and working dilutions

Antibody	Category number	Manufacturer	Dilution
HDAC1 (D5C6U) XP® Rabbit mAb	34589	Cell Signaling Technology	1:1000
HDAC2 (D6S5P) Rabbit mAb	57156	Cell Signaling Technology	1:1000
HDAC3 (D2O1K) Rabbit mAb	85057	Cell Signaling Technology	1:1000
HDAC4 (D15C3) Rabbit mAb	7628	Cell Signaling Technology	1:2000
HDAC6 (D2E5) Rabbit mAb	7558	Cell Signaling Technology	1:2000
Anti-rabbit IgG, HRP-linked Antibody	7074	Cell Signaling Technology	1:3000
Anti-mouse IgG, HRP-linked Antibody	7076	Cell Signaling Technology	1:3000
Anti-β-Actin (ACTB) Antibody	A5441	Sigma-Aldrich	1:5000
Acetyl-Histone H3 (Lys9) Rabbit PolyAb	29133-1-AP	Proteintech	1:2000
Acetyl-Histone H3 (Lys27) Rabbit RecAb	82902-1-RR	Proteintech	1:2000
Histone H3 Rabbit PolyAb	17168-1-AP	Proteintech	1:2000
ATF5 Recombinant Rabbit Monoclonal Antibody (SD2099)	MA5-32365	Invitrogen	1:2000
TRIB3 Polyclonal Antibody	PA5-15480	Invitrogen	1:1000
INHBE Polyclonal Antibody	PA5-51101	Invitrogen	1:1000
