# Supplementary material for: Responses to Nei-Xiao-Luo-Li-San in Toledo Diffuse Large B-Cell Lymphoma Cells: Potential Involvement of ATF5- and HDAC-Associated Epigenetic Changes
Source: Biomedicines. 2026 Sep 12;14(9):2052. doi: 10.3390/biomedicines14092052 (PMC13604743; doi:10.3390/biomedicines14092052)
Supplement: Supplementary file 1 [file biomedicines-14-02052-s001.zip › Supplementary Document S1 Generation of stable ATF5-overexpression and knockdown Toledo cell lines_v4.pdf]

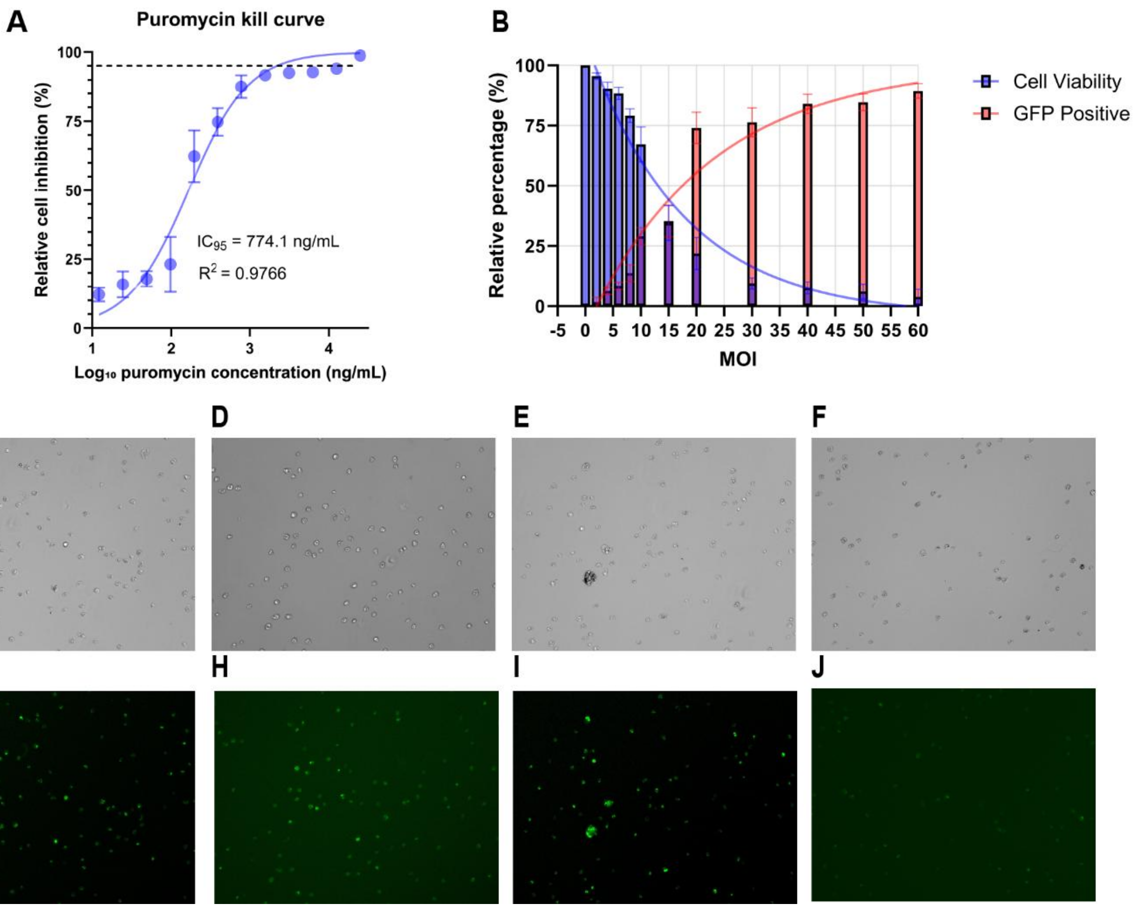

(A) Puromycin killing curve establishing the IC_{95} concentration for selection. (B) MOI optimization using the empty control vector. (C–F) Brightfield images of OE-NC, sh-NC, OE-ATF5, and sh-ATF5 groups. (G–J) Corresponding GFP fluorescence images indicating successful transduction.

To establish stable transduction conditions, a puromycin killing curve was first performed and the value of IC95 was determined to be 774.1 ngmL−1 (Fig. A). Subsequently, this concentration was used for the selection of antibiotics following lentiviral infection. A control empty vector was used to optimize the multiplicity of infection (MOI), and an MOI of 15 was selected as optimal (Fig. B).

After transduction, strong GFP fluorescence signals were observed in Toledo cells across groups under the GFP channel, confirming successful infection (Fig. C–J). OE-NC and sh-NC denote negative control cells transduced with empty overexpression or scrambled shRNA vectors, respectively, while OE-ATF5 and sh-ATF5 represent ATF5-overexpressing and ATF5-knockdown cells.
