# Supplementary material for: Responses to Nei-Xiao-Luo-Li-San in Toledo Diffuse Large B-Cell Lymphoma Cells: Potential Involvement of ATF5- and HDAC-Associated Epigenetic Changes
Source: Biomedicines. 2026 Sep 12;14(9):2052. doi: 10.3390/biomedicines14092052 (PMC13604743; doi:10.3390/biomedicines14092052)
Supplement: Supplementary file 1 [file biomedicines-14-02052-s001.zip › Supplementary Document S2 Quality control reports for NXLLS herbal materials.pdf]

江阴天江药业有限公司化验室

JIANGYIN TIANJIANG PHARMACEUTICAL CO. , LTD. LABORATORY

NO. 1 XIN SHEN ROAD， JIANGSU， CHINA 214434

CERTIFICATE OF ANALYSIS

品名 PRODUCT NAME：Nei Xiao Luo Li San (Max's Formula)

生产厂家 PRODUCER：TIANJIANG PHARMACEUTICAL CO. ，LTD.

生产日期 DATE OF PRODUCTION： 2020/11/6 批号BATCH NO.： 907272000027

报告日期 DATE OF REPORT： 2020/11/11 编码 Code： B2022C

检验依据：CORRESPONDING TO CHINESE PH. AND TIANJIANG PHARM. SPECIFICATION

检验项目DETERMINATION		标准SPECIFICATION	结果RESULT
外观 Appearance：		黄棕色颗粒Yellow brown granule.	Conform
品种 Identification：		TLC	Conform
水分 Moisture：		≤8. 0%	3.9%
溶化性 Solubility：		Soluble in hot water	Conform
细菌 Microbiological：		(Less than 1000 cfu /g,Culture vessels)	<10cfu /g
霉菌 Fungal count：		(Less than 100 cfu /g,Culture vessels)	<10cfu /g
大肠埃氏菌 Escherichia coli：		(absent in 1g,In vitro Culture)	Negative
重金属 Heavy metals:			
	Pb：	(Less than 10mg/kg)	0.020 mg/kg
	Cd：	(Less than 1.0mg/kg)	Negative
	As：	(Less than 5mg/kg)	0.227 mg/kg
	Hg：	(Less than 0.2mg/kg)	0.001 mg/kg

检验结果 Final result: 符合规定 Conform

江阴天江药业有限公司 检测报告专用章

化验员 ANALYST： 孔磊 刘黔录

复核人 COLLATOR： [illegible]

质保部经理MANAGER： [illegible]

Bat # 26618 B2022C-90s Sku: 4927

Concentrated Nei Xiao Luo Li San
(Max's Formula) 90g Powder (in bottle)

907272000027
