# Supplementary material for: Responses to Nei-Xiao-Luo-Li-San in Toledo Diffuse Large B-Cell Lymphoma Cells: Potential Involvement of ATF5- and HDAC-Associated Epigenetic Changes
Source: Biomedicines. 2026 Sep 12;14(9):2052. doi: 10.3390/biomedicines14092052 (PMC13604743; doi:10.3390/biomedicines14092052)
Supplement: Supplementary file 1 [file biomedicines-14-02052-s001.zip › Supplementary Document S3 TLC identification.pdf]

TIAN JIANG

Work performed at:
Testing Center of Jiangyin Tianjiang Pharmaceutical Co., Ltd.
(ISO-17025 Laboratory)
No.1, Xin Sheng Road, High-Tech Zone, Jiangyin, Jiangsu, China
+86-510-86408692
tjgjywb@tianjiang.com
www.tianjaing.com

TLC test record: Nei Xiao Luo Li San (Max's Formula) 907272000027

Thin-Layer Chromatography with Photo-Documentation

BAI ZHI
(白芷)

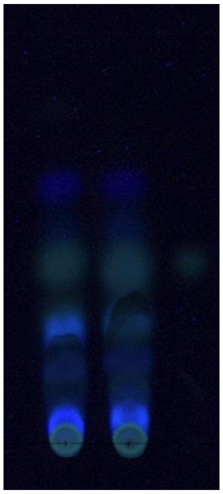

Result: PASS

JIE GENG
(桔梗)

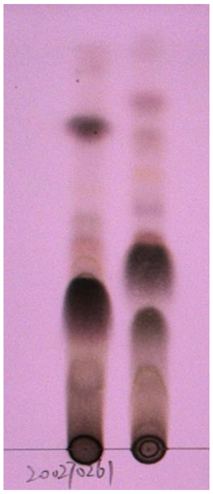

Result: PASS

MU LI
(牡蛎)

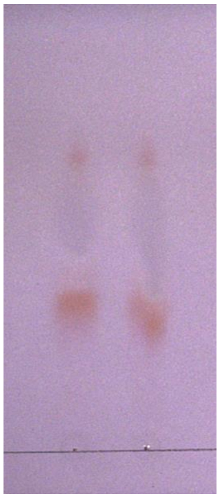

Result: PASS

SHU DA HUANG
(熟大黄)

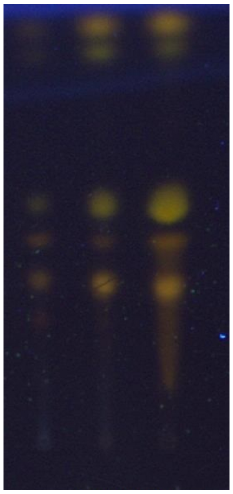

Result: PASS

TIAN HUA FEN
(天花粉)

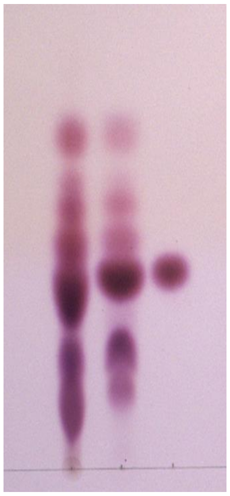

Result: PASS

XIA KU CAO
(夏枯草)

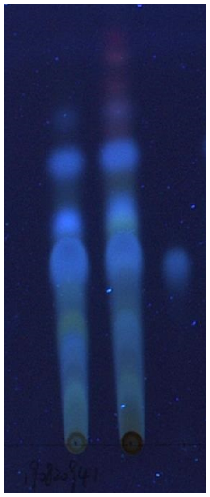

Result: PASS

XUAN SHEN
(玄参)

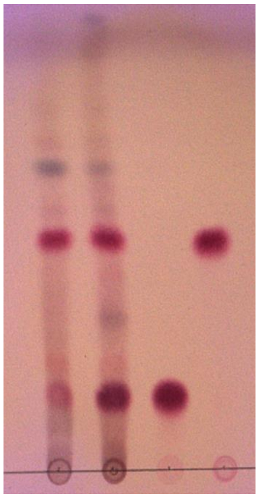

Result: PASS

ZHE BEI MU
(浙贝母)

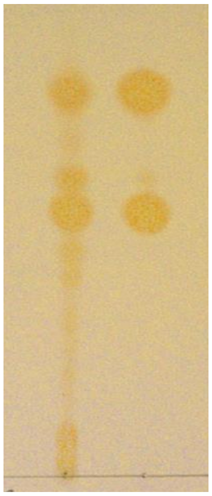

Result: PASS
