# Supplementary material for: Responses to Nei-Xiao-Luo-Li-San in Toledo Diffuse Large B-Cell Lymphoma Cells: Potential Involvement of ATF5- and HDAC-Associated Epigenetic Changes
Source: Biomedicines. 2026 Sep 12;14(9):2052. doi: 10.3390/biomedicines14092052 (PMC13604743; doi:10.3390/biomedicines14092052)
Supplement: Supplementary file 1 [file biomedicines-14-02052-s001.zip › Supplementary Document S4 HPLC fingerprint similarity analysis of NXLLS batches_V5.pdf]

Batch A-3

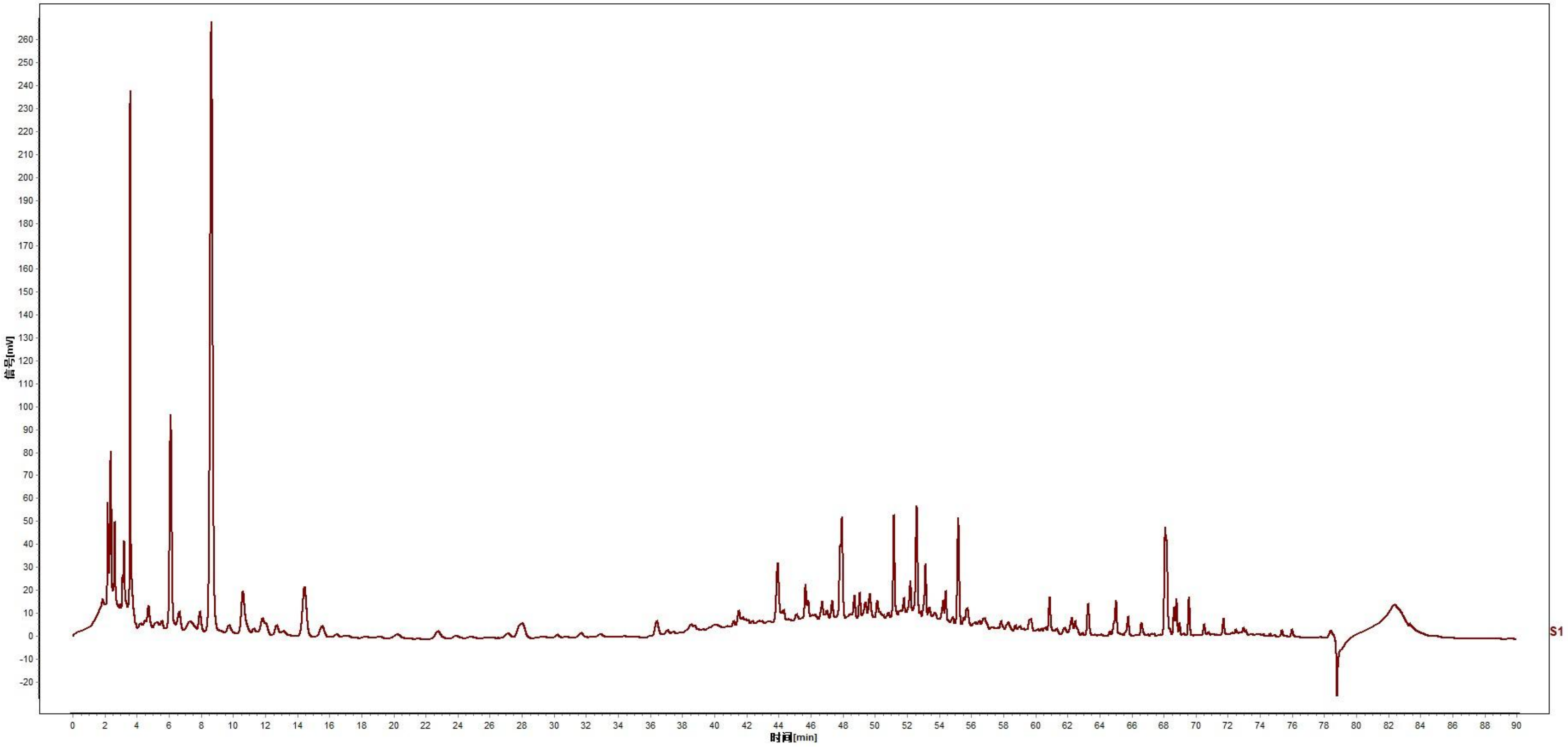

Batch A-2

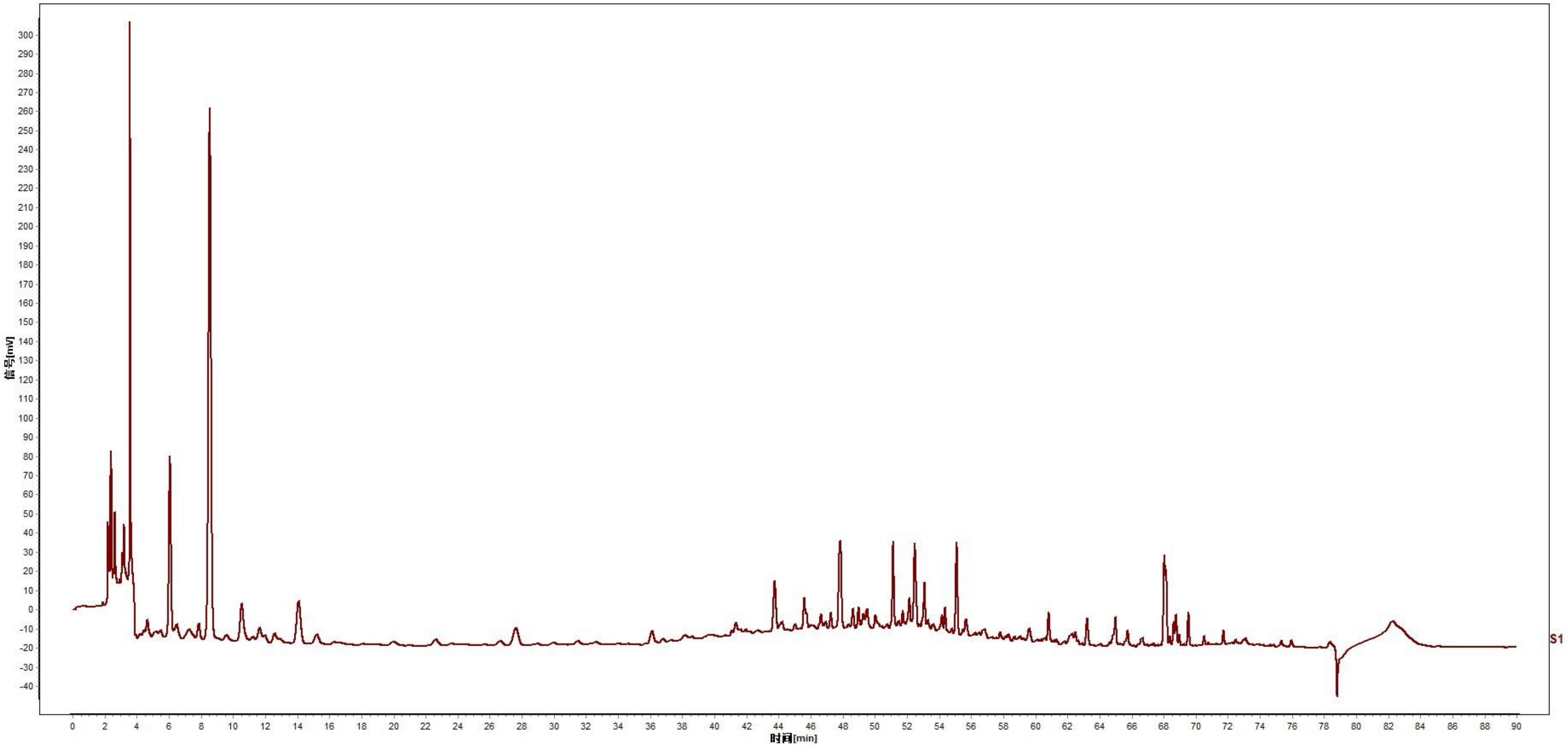

Batch A-1

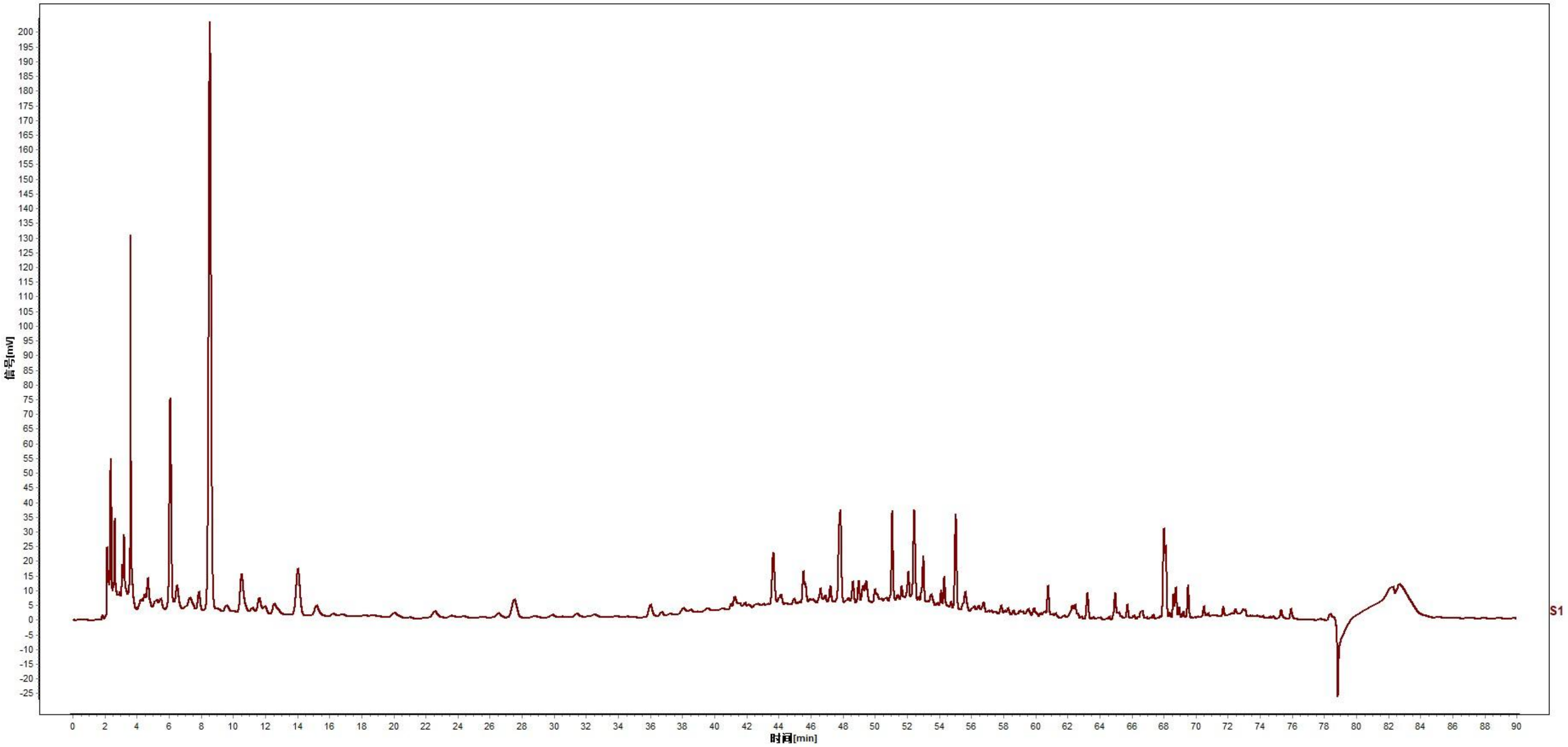

Batch B-3

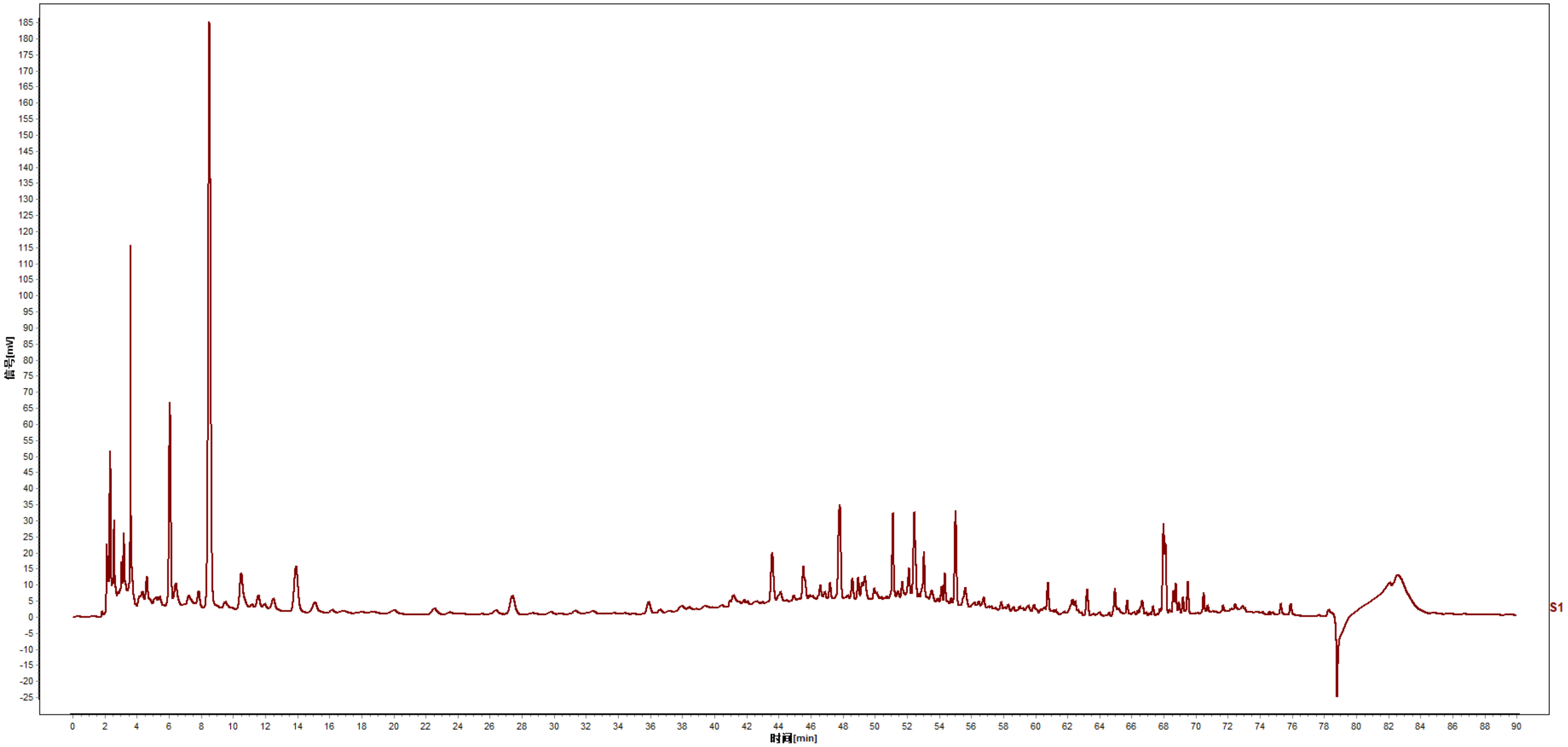

Batch B-2

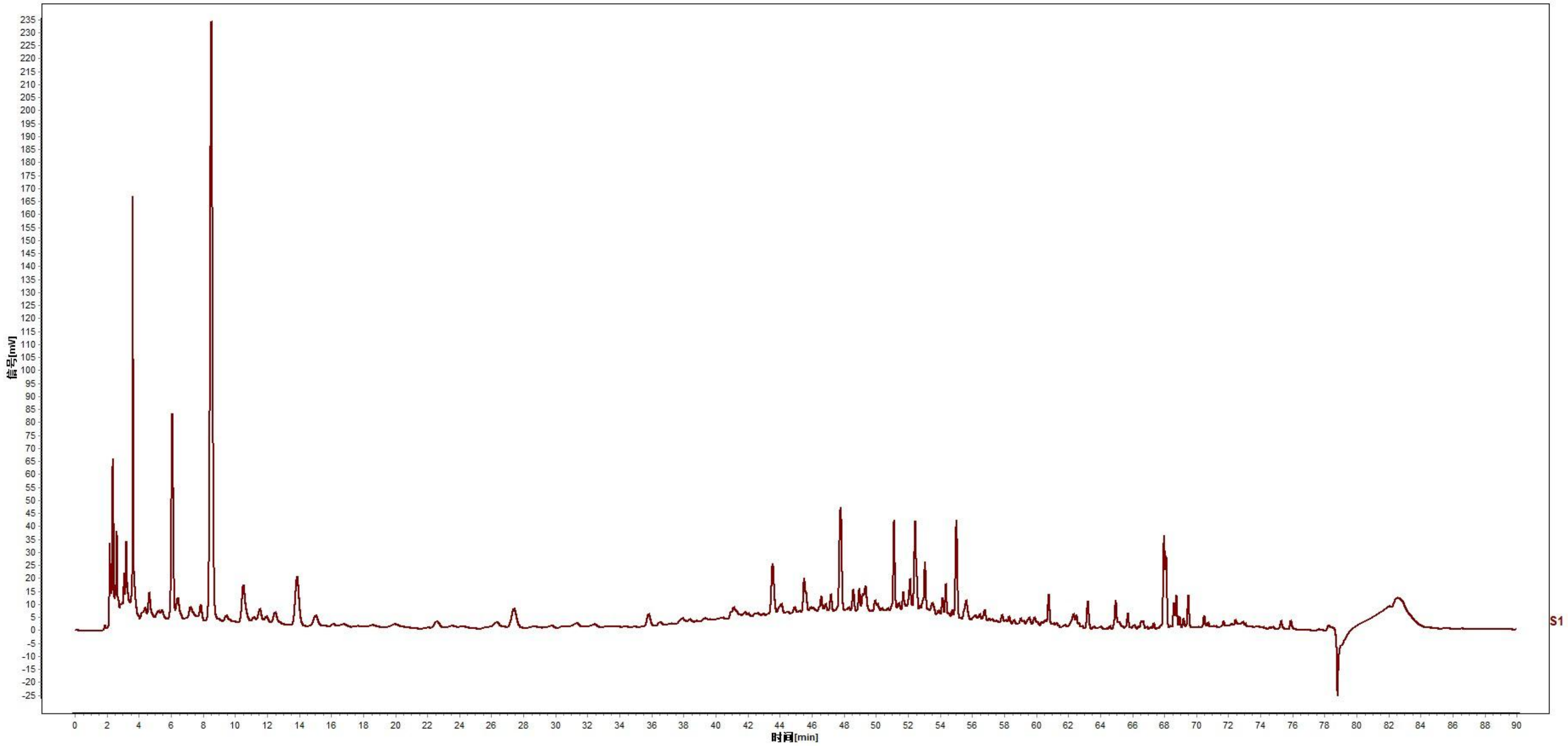

Batch B-1

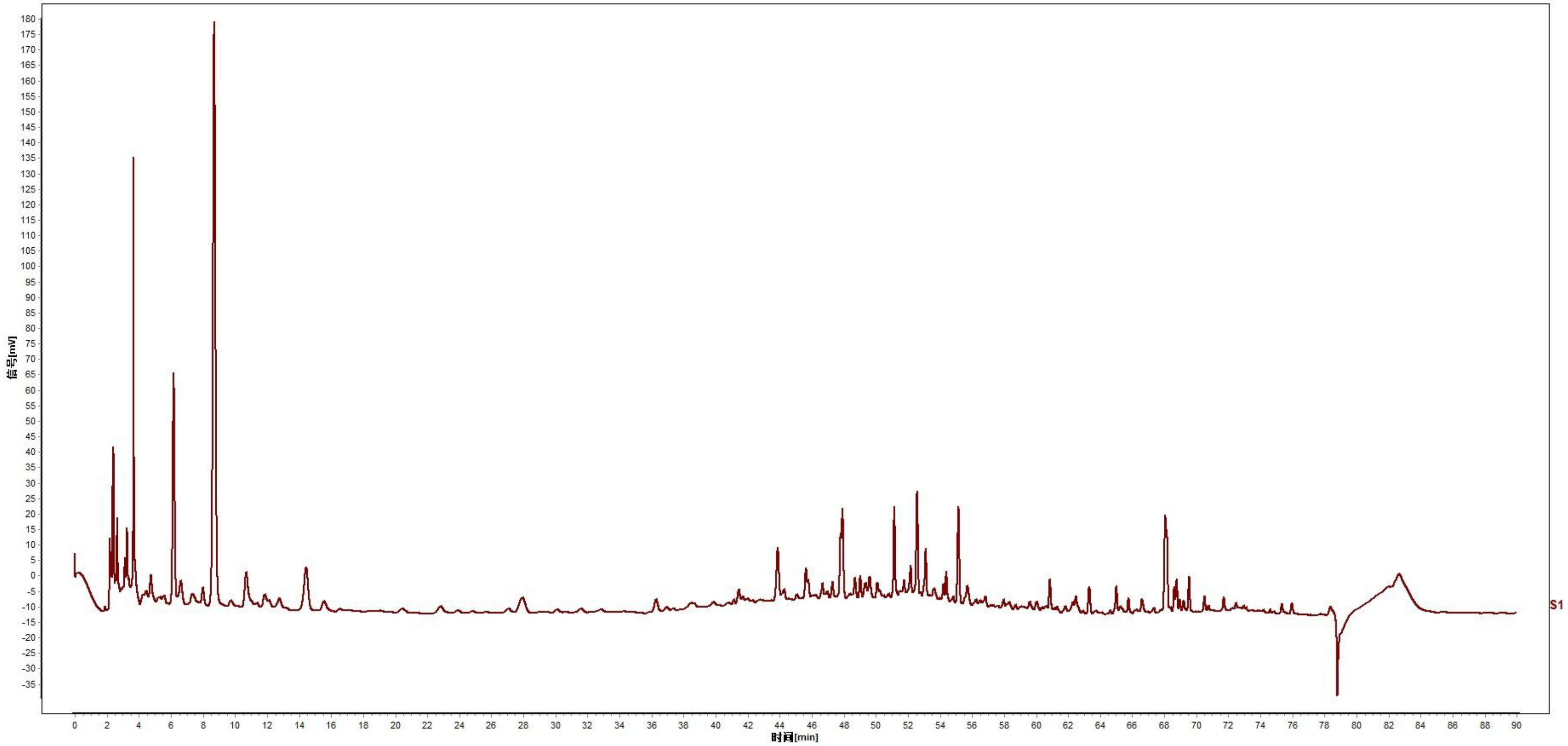

Batch C-3

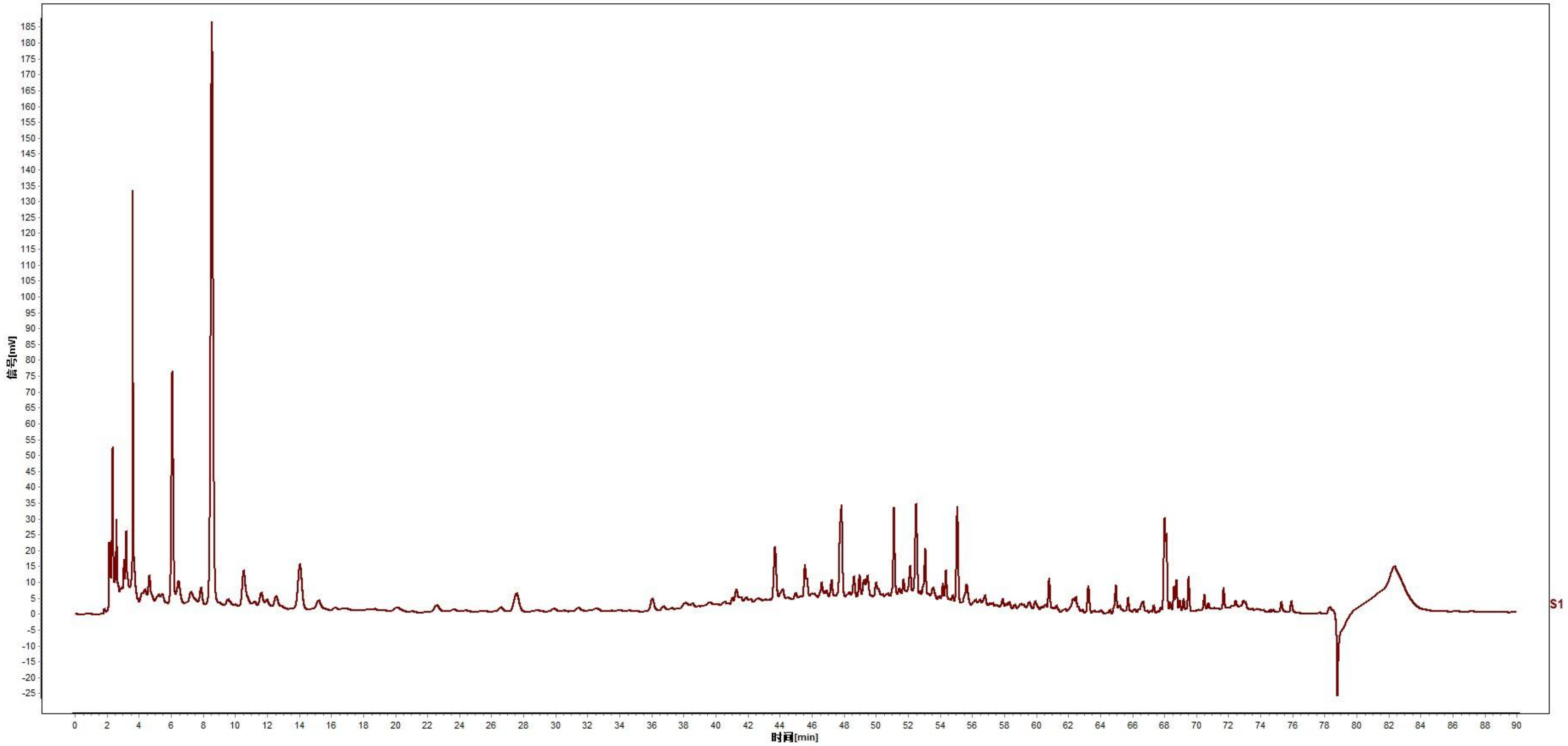

Batch C-2

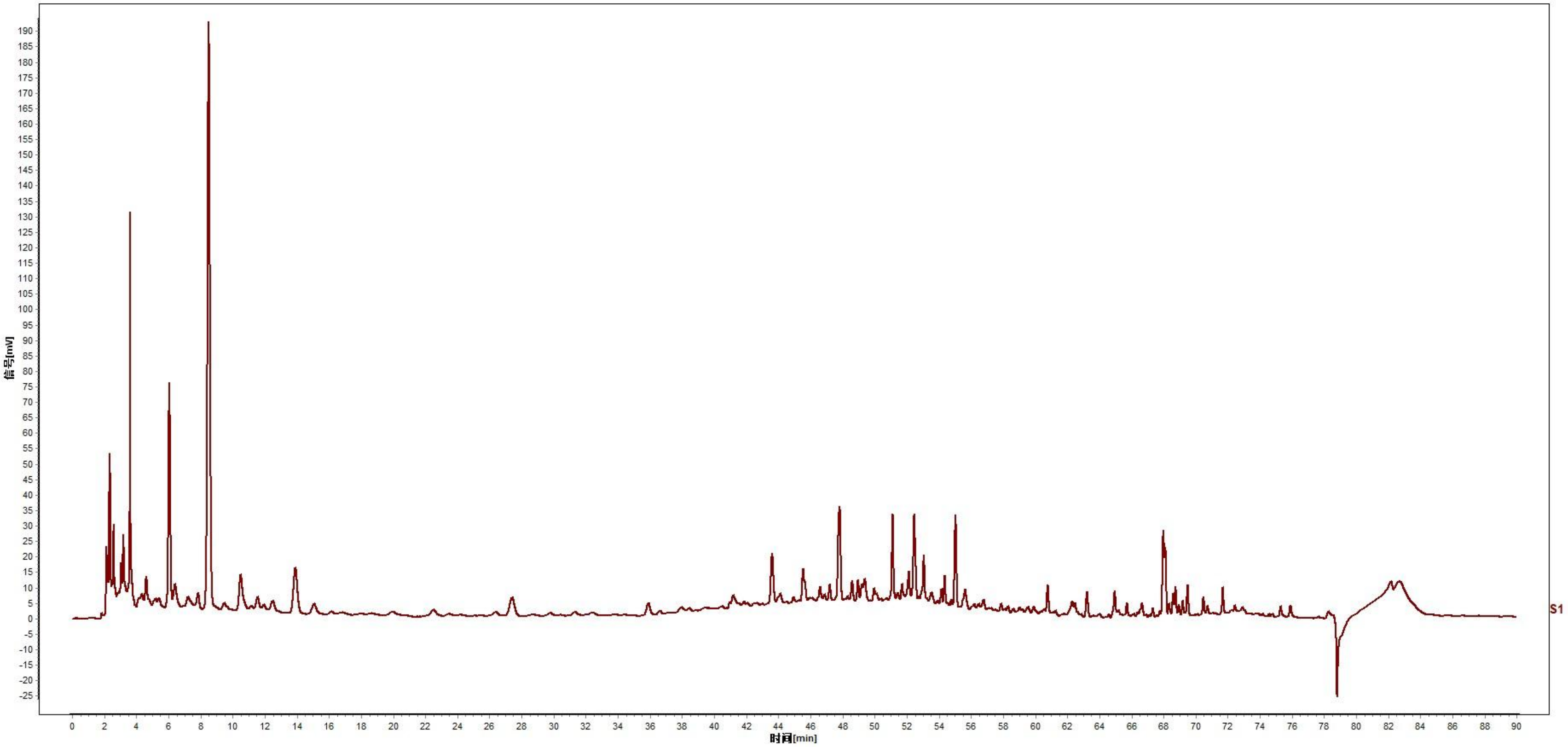

Batch C-1

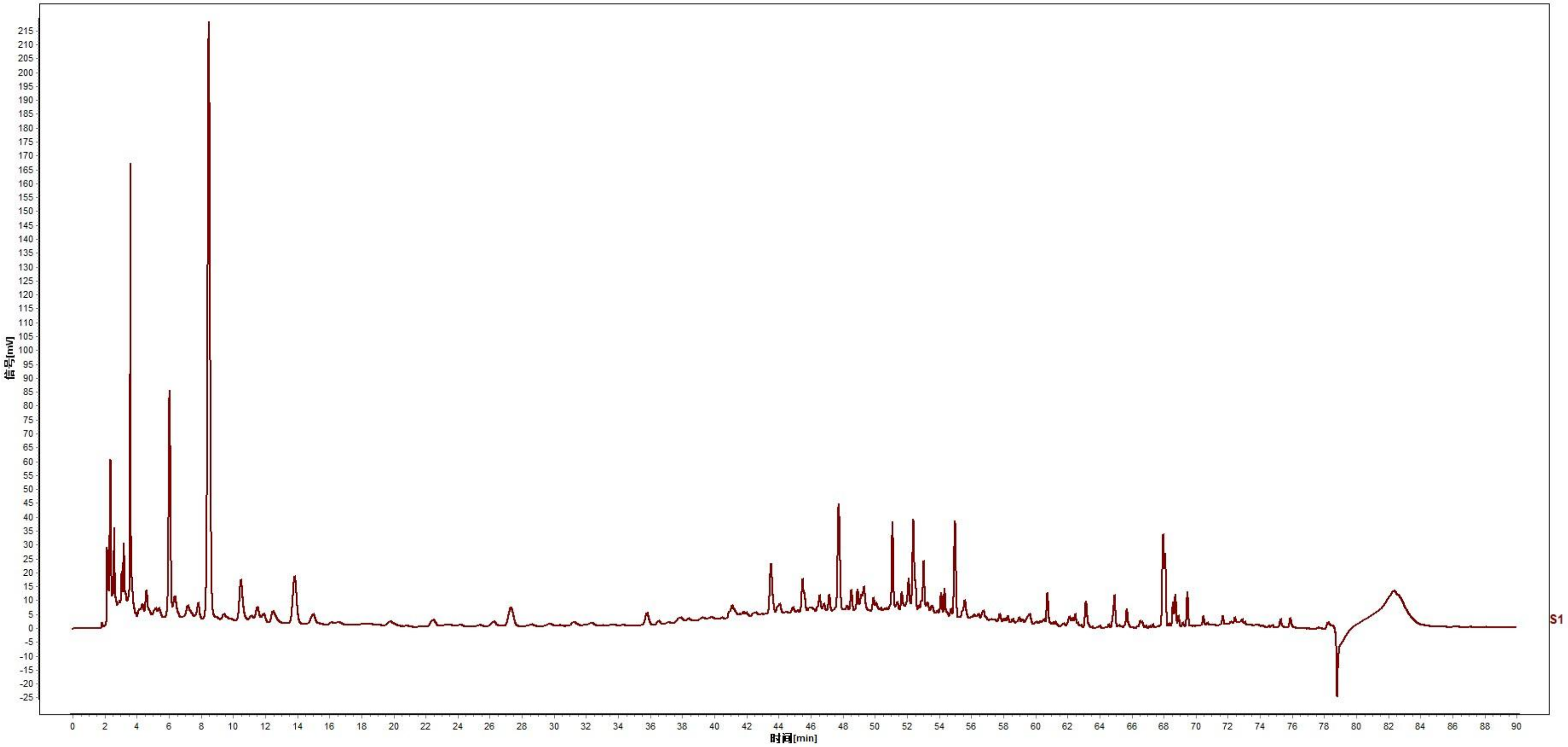

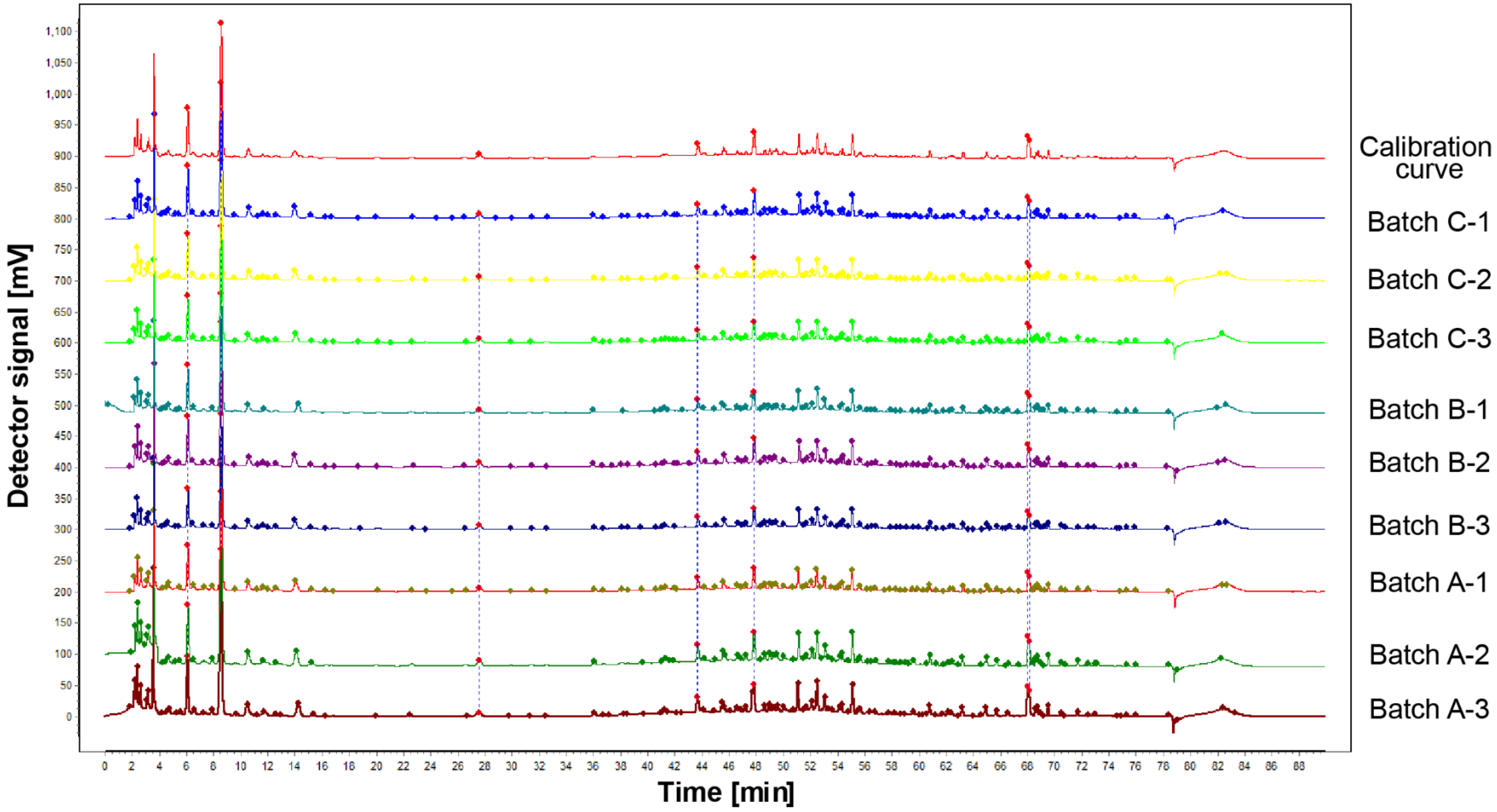

Similarity analysis of HPLC fingerprints across different NXLLS batches. Batch A represents the primary batch used in this study. Three independent samples were analyzed for each batch. The top panel shows the calibration curve generated based on nine samples.

	Similarity analysis results									
	Batch A-3	Batch A-2	Batch A-1	Batch B-3	Batch B-2	Batch B-1	Batch C-3	Batch C-2	Batch C-1	Reference fingerprint chromatogram
Batch A-3	1	0.958	0.990	0.869	0.950	0.933	0.921	0.936	0.917	0.964
Batch A-2	0.958	1	0.952	0.929	0.931	0.936	0.910	0.953	0.938	0.960
Batch A-1	0.990	0.952	1	0.943	0.886	0.950	0.892	0.941	0.922	0.952
Batch B-3	0.869	0.929	0.943	1	0.961	0.973	0.977	0.926	0.881	0.969
Batch B-2	0.950	0.931	0.886	0.961	1	0.943	0.945	0.959	0.931	0.933
Batch B-1	0.933	0.935	0.950	0.971	0.943	1	0.915	0.925	0.962	0.973
Batch C-3	0.921	0.910	0.892	0.977	0.945	0.915	1	0.989	0.975	0.931
Batch C-2	0.936	0.953	0.941	0.926	0.959	0.925	0.989	1	0.966	0.943
Batch C-1	0.917	0.938	0.922	0.881	0.931	0.962	0.975	0.966	1	0.971
Reference fingerprint chromatogram	0.964	0.960	0.952	0.969	0.933	0.973	0.931	0.943	0.971	1

The reference fingerprint chromatogram was generated from the average chromatographic profiles of all nine samples. Pairwise similarity values among the nine samples ranged from 0.869 to 0.990. The mean intra-batch similarity was 0.967, exceeding the mean inter-batch similarity of 0.929. Similarities between the individual chromatograms and the reference fingerprint ranged from 0.931 to 0.973. These results indicate high overall chromatographic similarity within batches, while some inter-batch variation remained. However, the minimum pairwise similarity value of 0.869 fell below the commonly referenced threshold of 0.90, indicating that some inter-batch chromatographic variation remained.
